# Beyond text: quantifying the impact of figure-guided retrieval in biomedical RAG systems

**DOI:** 10.3389/frai.2026.1861611

**Published:** 2026-09-29

**Authors:** Raghav Kishore Venkatesh, G. Sudhakaran, C. U. Om Kumar

**Affiliations:** 1 School of Electronics Engineering, Vellore Institute of Technology, Chennai, India; 2 School of Computer Science and Engineering, Vellore Institute of Technology, Chennai, India

**Keywords:** BioASQ-13b, biomedical information retrieval, figure-first retrieval, multimodal retrieval, PubMed central, query-focused summarization, Retrieval-Augmented Generation

## Abstract

**Introduction:**

Retrieval-Augmented Generation (RAG) has been established as a reliable paradigm for grounding large language model (LLM) outputs in factual evidence, acting as a safeguard in high-stakes domains such as biomedicine, where hallucination carries significant consequences. However, existing biomedical RAG systems rely predominantly on text-only corpora, overlooking the rich semantic information encoded in figures, charts, and other visual evidence. In this work, we investigate whether incorporating figure-based retrieval methods into a hybrid RAG pipeline meaningfully improves generation quality and retrieval effectiveness in the biomedical domain.

**Methods:**

We propose Figure-First Hybrid RAG (FFHRAG), a multi-stage retrieval architecture integrating multi-channel figure retrieval with hybrid text retrieval. Our architecture employs a **figure-first** discovery mechanism that retrieves relevant visual evidence, and utilizes it as a guiding signal for subsequent text selection. This strategy eliminates the need for complex embedding alignment, offering a **low-overhead** system that avoids **cross-modal embedding noise** at the final text chunk selection stage. The system is evaluated zero-shot on summary-type questions derived from BioASQ-13b, against a corpus spanning over 1.1 million PubMed Central text chunks and 82,000 figure-captions.

**Results:**

A progressive, multi-seed six-variant ablation study demonstrates that while the text-only baseline components establish a strong retrieval foundation—BM25 retriever yielding a Recall@10 of 0.7322 ± 0.0154 and the Cross-Encoder reranking securing an MRR of 0.8331 ± 0.0018, our proposed framework introduces isolated, targeted enhancements. Specifically, the localized PMCID-level boosting module refines retrieval ordering to an MRR of 0.8358 ± 0.0018, while prompt-level figure caption injection substantially enriches downstream generation quality, driving ROUGE-1 from 0.4068 ± 0.0037 to 0.4137 ± 0.0036 and ROUGE-L from 0.2623 ± 0.0018 to 0.2678 ± 0.0016 over the strong text-only baseline.

**Discussion:**

Our findings suggest that figures represent an underutilized retrieval modality in biomedical RAG systems, and that even lightweight figure-aware boosting mechanisms can produce measurable gains in answer quality without requiring multimodal fusion at the embedding level.

## 1 Introduction

Biomedical literature has expanded at a rate that fundamentally outmatches the human capacity for manual knowledge synthesis. PubMed, the primary repository for biomedical and life science publications, currently possesses over 36 million articles, with more than a million new entries added every year, averaging approximately 2,700 new articles every single day (Jin et al., 2024). This volume makes manual synthesis practically infeasible; studies estimate that answering a single complex medical query through traditional literature search requires up to four expert hours (Jin et al., 2023b), making it almost impossible for any individual to remain comprehensively informed across even a narrow subfield. This bottleneck has shifted automated synthesis from a technical convenience to a clinical necessity. Early automated approaches to biomedical text synthesis followed extractive or abstractive paradigms. Extractive methods produced semantically disjointed summaries by concatenating isolated sentences without preserving logical coherence. Abstractive models, although capable of producing semantically fluent text, frequently hallucinated, producing factually incorrect statements that contradicted source documents. Both paradigms were additionally restricted by the token length limitations of early neural architectures, necessitating the truncation of lengthy, multi-section documents typical of biomedical literature (Celikten and Onan, 2025; Xie et al., 2023).

Significant advancements were made to the existing Information Retrieval and abstractive summarization systems through the introduction of Large Language Models (LLMs). Using dense retrieval techniques like BioBERT and PubMedBERT, queries and documents can now be represented in the same semantic embedding space, allowing for the identification of domain-specific words that are not available with older methods of retrieval based solely on the superficial level of language (Amugongo et al., 2025). LLMs used self-attention mechanisms to handle long-range dependencies in biomedical documents, creating coherent, abstract summaries that preserved the context instead of merely piecing information together. Their prompt-based flexibility also made it possible to adapt to different task needs without having to retrain, which was a major change from the rigid classification-based systems that existed before (Celikten and Onan, 2025). Even with these improvements, standalone LLMs possessed critical limitations that restricted their deployment in biomedical settings. LLMs are especially prone to hallucination, making up false claims and presenting them with unwarranted confidence. This is a critical issue that has direct safety implications for both research and clinical decision-making (Amugongo et al., 2025; Liu et al., 2025). Additionally, LLMs utilize static parametric memory that is frozen at the end of their training, rendering them incapable of answering about new research findings, updated treatment protocols, or new clinical guidelines in a field which is rapidly evolving. Furthermore, LLMs encode knowledge implicitly across model parameters with no traceable citation pathway, making it impossible for clinicians to verify the provenance of a recommendation which is a fundamental requirement of evidence-based medicine (Amugongo et al., 2025; Liu et al., 2025). These limitations collectively motivate the adoption of Retrieval-Augmented Generation, which anchors model outputs in dynamically retrieved, verifiable external evidence rather than relying on internalized parametric knowledge (Amugongo et al., 2025; Liu et al., 2025).

Retrieval-Augmented Generation (RAG) addresses these constraints by decoupling knowledge storage from model parameters. External knowledge corpora are indexed as dense vector representations, and at inference time, the top semantically relevant chunks are retrieved and concatenated with the query as grounding context for the LLM generator. By conditioning generation on dynamically retrieved, verifiable evidence rather than static parametric memory, RAG effectively suppresses the generation of baseless assertions (Amugongo et al., 2025; Liu et al., 2025). A meta-analysis of clinical RAG implementations reported a statistically significant 1.35 odds ratio improvement in task performance over standalone LLMs (Liu et al., 2025). Unlike in fine-tuning approaches, RAG ensures that models have constant access to the latest research and guidelines without any need for retraining. The key advantage here is the ability to create an unambiguous chain of citation since the results of an analysis are always grounded in the sources retrieved, which is important for evidence-based medicine. In the biomedical domain, RAG systems leverage domain-adapted retrievers such as PubMedBERT and BioBERT to retrieve from authoritative repositories like PubMed Central, capturing specialized clinical synonymy and terminology that general-purpose encoders systematically mishandle (Amugongo et al., 2025).

While RAG has proven to be effective for biomedical NLP, existing systems remain predominantly text-centric in their retriever design. Most implementations focus exclusively on textual knowledge retrieval, with limited exploration of truly integrated multimodal approaches (Liu et al., 2025; El-Enen et al., 2025). This text-centric approach ignores the reality of the biomedical domain, where visual evidence is not just a mere supplement to the text. Figures, charts, and diagrams in biomedical literature frequently encode primary experimental evidence that either complements or cannot fully be conveyed through textual descriptions alone (Jin et al., 2023b). Despite this high information density, integrated frameworks that leverage visual and textual signals together remain underdeveloped, and the specific contribution of figure-based retrieval signals to RAG pipeline effectiveness has not been systematically studied (Amugongo et al., 2025). In this work, we address this gap directly: we propose Figure-First Hybrid RAG (FFHRAG), a modular multi-stage retrieval architecture designed as a generalizable orchestration framework for text-visual cross-linking. FFHRAG establishes an architectural design pattern that optimizes how distinct textual and visual modalities interact. We conduct a progressive ablation study across six system variants to empirically quantify the impact of figure-guided retrieval on both retrieval effectiveness and generation quality in the biomedical domain.

### 1.1 Contributions

1. **Figure-First Retrieval framing:** We introduce a figure-first retrieval architecture that integrates a four-channel figure retrieval: combining dense PubMedBERT caption embeddings, CLIP-based caption and image encoders, and sparse BM25 search, with a cross-modal boosting mechanism (Figureboost) that propagates figure relevance signals into text retrieval. This architecture propagates figure relevance signals directly into text retrieval scoring without forcing textual and visual features into a shared cross-modal embedding space or relying on late-stage embedding fusion. By maintaining separate retrieval pathways, it offers a low-overhead approach that completely eliminates cross-modal representation noise at the final text chunk selection stage.
2. **Generator benchmarking framework:** We conduct a zero-shot generator benchmarking study across six large language models using inputs retrieved from our FFHRAG, on a specially curated dataset of 650 biomedical summary questions, taken from the BioASQ-13b dataset, establishing a reproducible generator selection methodology and baseline for future biomedical RAG research under standard retrieval conditions.
3. **Retriever ablation study and metric framing:** We present a progressive six-variant ablation study isolating the contribution of each pipeline component, evaluated through a multi-dimensional framework spanning retrieval quality, generation quality, a novel set of domain-level metrics, as well as figure-specific metrics, representing a novel and rigorous evaluation study reproducible by future biomedical RAG benchmarking.
4. **Hyperparameter sensitivity analysis framing:** We conduct a systematic sensitivity analysis across four key hyperparameters: MMR diversity weight, Figureboost scoring mechanism and thresholding, and RRF fusion constant, demonstrating that maximum metric variation across sweeps remains below 0.005, confirming that performance gains are robust and not artifacts of parameter tuning.
5. **Robust gains across metrics:** We provide empirical evidence demonstrating that the targeted injection of cross-modal context yields distinct, statistically significant enhancements to abstractive generation quality. While the underlying text infrastructure establishes a high baseline floor, our figure-first components drive final generation metrics to a ROUGE-1 of 0.4137 ± 0.0036 and a ROUGE-L of 0.2678 ± 0.0016 over the text-only baseline, while preserving a highly precise final retrieval ranking with an MRR of 0.8358 ± 0.0018.

This study is organized as follows: Section 2 describes the current methods used in Biomedical RAG systems. Section 3 discusses the materials, proposed framework, and methods used in this study. Results and discussion are explained in Section 4. The conclusion and future scope are discussed in Section 5.

## 2 Related work

Biomedical question answering has been extensively studied through the BioASQ challenge, a long-standing benchmark series evaluating systems on large-scale semantic indexing and generation of ideal answers from PubMed-derived questions (Tsatsaronis et al., 2015). The thirteenth edition, BioASQ 13b, comprised a training set of 5,389 questions, including 1,283 summary-type questions, and a test set of 340 expert-annotated questions evaluated via MAP for document retrieval, F1 for snippet retrieval, and ROUGE alongside manual expert scoring for ideal answers. The 46 teams that were competing utilized a variety of methods, including traditional BM25 and dense retrieval models like BGE-M3 and MiniLM, which were further improved upon by re-ranking techniques. Additionally, systems utilizing Retrieval-Augmented Generation (RAG) frameworks frequently employed large language models (LLMs) like Llama, GPT, and Mistral for answer generation, with zero-shot prompting and ensemble methods being commonly implemented by participating teams (Nentidis et al., 2026).

To understand the selection of this benchmark, we need to compare its objectives and structure with other prominent biomedical evaluation datasets and repositories, such as PubMedQA and MIMIC. PubmedQA evaluates clinical, as well as biomedical question answering using question-context-answer triplets, which are derived strictly from PubMed abstracts (Jin et al., 2019). However, it is structurally optimized for a final decision of “yes”, “no”, or “maybe” from the models evaluated, along with a descriptive explanation. The MIMIC repositories compile longitudinal, de-identified electronic health records, which feature clinical notes, radiology reports, and structured measurements (Johnson et al., 2016). Evaluation targets within MIMIC focus on clinical chart and note extraction, such as transforming unstructured text into organized clinical data, extracting precise medications, generating reports centered on a single patient's continuous clinical record, or resolving closed-ended visual queries based on a single image-report pair. While exceptional for testing single-document classification or localized patient data extraction, these alternative frameworks do not align with the requirements of our task, which is related to retrieving information from multiple documents and synthesizing a short paragraph answer. A BioASQ task-b summary question cannot be resolved by a single abstract or a localized clinical chart. Instead, its task definition explicitly mandates that systems search, retrieve, and combine fragmented evidence across large-scale document repositories, text snippets, databases, and ontologies. The system must then distill these heterogeneous signals into a coherent, paragraph-sized summary capped at a maximum of 200 words that mirrors an “ideal” response authored by a peer biomedical scientist. This distinct requirement for open-ended, multi-document context generation makes the BioASQ summary split uniquely suited to evaluate the structural capabilities of our figure-first pipeline.

Beyond the official competition, several works independently evaluate retrieval and generation pipelines on BioASQ training subsets, the evaluation paradigm we adopt in this work. BioRAGent employs a multi-agent, tool-augmented RAG system to navigate intricate biomedical data structures. The authors curated a specialized subset of approximately 200 questions from BioASQ task B, specifically targeting the complex associations between genes, diseases, and phenotypes to benchmark reasoning accuracy against state-of-the-art models as a supplementary analysis (Bi et al., 2025). Gao et al. (2024) demonstrated that hierarchical retrieval combining coarse BM25 filtering with fine-grained dense ensemble retrieval substantially improves summary answer quality on BioASQ 12, establishing retrieval depth as a key driver of generation performance, a notion which has further been proved in our work. However, across these works, the incorporation of figures in the retrieval pipeline has been minimal.

Recent work in multimodal retrieval has explored diverse strategies for incorporating visual signals into document retrieval pipelines, broadly falling into two paradigms. The first paradigm treats entire document pages as visual retrieval units, adapting advanced text-based late-interaction models—such as ColBERT, which evaluate fine-grained token-level embeddings rather than compressing an entire passage into a single global vector—into a multimodal architecture. Within this layout, ColPali encodes full document pages as multi-vector image patch embeddings using a Vision Language Model, employing a visual adaptation of this late-interaction mechanism to match text query tokens directly against visual document representations (Faysse et al., 2025). Beyond single-vector dense architectures, text-centric retrieval frameworks have increasingly leveraged multi-vector late-interaction matching paradigms to preserve granular semantic details in the biomedical domain. For instance, Makhija and Tanwani (2025) combined sparse and dense retrieval channels with a ColBERT-based re-ranking step to refine candidate pools for downstream generation on the BioASQ benchmark. Similarly, Rivera and Menolascina (2025) paired a lightweight ModernBERT bi-encoder with a fine-tuned ColBERTv2 retriever on the MIRAGE benchmark, demonstrating that token-level maximum-similarity operators are uniquely capable of preserving critical clinical negation context that global vector compressions regularly dilute. Furthermore, specialized multi-vector architectures like ClinicalEncoder26AM introduce diagnosable late-interaction models aligned to clinical reference spaces to optimize multilingual entity recall over extensive text windows (Remy, 2026). Other works such as VisRAG run parallel visual and textual RAG pipelines over page-level embeddings, fusing their reasoning chains via consistency-constrained modality alignment (Yu et al., 2025), and MRAG-Suite benchmarks dual-encoder CLIP and DPR retrieval by serializing independently retrieved images and text chunks as parallel prompt inputs (Ji et al., 2025).

The second paradigm uses input images as retrieval queries: RAMM retrieves biomedical image-text pairs from PMC patient case reports via image-text contrastive similarity (Yuan et al., 2023), while MMed-RAG employs domain-aware routing of input medical images to specialized visual retrievers to fetch relevant clinical reports (Xia et al., 2025), and RULE further calibrates retrieved context volume using factuality risk control over image-to-text retrieval results (Xia et al., 2024). Closer to the biomedical literature setting, AlzheimerRAG generates image captions via LLaVA and fuses cross-modal embeddings to retrieve multimodal content from PubMed articles (Lahiri and Hu, 2025), and (Trabucco et al., 2023) index figure modality metadata in Apache Lucene to enable visual modality filtering alongside text queries over PMC documents.

These vision-based architectures demonstrate robust capabilities in native document layout understanding, spatial geometry mapping, and graphic tracking. By evaluating pages or images as complete visual entities, they successfully preserve embedded data tables, charts, and structural cross-references that text-only parsers regularly omit. However, computing pixel-level multi-vector patch matrices across extensive digital libraries at query time can introduce substantial processing overhead and storage demands. FFHRAG offers a complementary, modular alternative designed for a low-overhead operational profile, achieving cross-modal grounding while maintaining a compact computational footprint. Instead of processing high-dimensional full-page image spaces at the query interface, FFHRAG isolates visual representation to an upstream multi-channel discovery layer. This maps figure relevance directly into text chunk boundaries using a metadata-level boost, bypassing cross-modal embedding noise during the final passage selection step while preserving generation accuracy within highly efficient resource boundaries.

## 3 Materials and methods

This section describes the architectural design and operational logic of our Figure-First Hybrid Retrieval-Augmented Generation (FFHRAG) system. We first formalize the task and characterize the evaluation dataset, followed by a detailed explanation of the FFHRAG pipeline.

### 3.1 System design and architecture

#### 3.1.1 Task formulation

The primary objective of FFHRAG is to perform figure-guided biomedical query summarization, specifically targeting queries that require the synthesis of evidence from disparate sources into a cohesive, paragraph-length answer. Given a natural language query *q*, the system retrieves a candidate set of textual contexts *C* from a large-scale biomedical corpus. Unlike standard retrieval pipelines, this process is explicitly conditioned on a set of visually and semantically relevant figures *F* identified in an upstream discovery stage. We formalize the generation of a grounded summary answer *A* as follows:

$$\begin{array}{l} A=\text{Generator}\left(q,\;C^{*}\left(q\right)\right) \end{array}$$ (1)

where C^*^(*q*) denotes the optimized context set passed to the generator, a ranked subset of text chunks whose selection is directly influenced by cross-modal visual signals, as described below. Equation 1 mirrors the standard RAG generation; the key departure from prior work lies entirely in how C^*^(*q*) is constructed, formalized in Equations 2, 3. The context set C^*^(*q*) is derived by scoring each candidate text chunk *c* through a figure-guided ranking function. For every chunk *c* in the fused retrieval pool, its relevance score is computed as:

$$\begin{array}{l} s\left(c,q\right)=\{\begin{array}{cc} f_{\text{boost}}\left(s_{\text{CE}}\left(c,q\right)\right) & \text{if}\;\text{PMCID}\left(c\right)\in B\left(q\right)\\ s_{\text{CE}}\left(c,q\right) & \text{otherwise} \end{array} \end{array}$$ (2)

where *s*_CE_(*c, q*) is the Cross-Encoder logit score of the text chunk, *f*_boost_ is the FigureBoost equation, and B(*q*) is the **Boosted PMCID set**—the set of PMCIDs which serve as identifiers of the top-ranked figures retrieved for query *q*. The piecewise formulation ensures that the score boosting mechanism only applies to chunks whose PMCIDs belong in the set B(*q*), meaning that at least one figure was retrieved by the figure retrieval pipeline from the same source document as the chunk. All remaining chunks whose PMCIDs do not belong in the boosted PMCID set, retain their base CE score.

The construction of C^*^(*q*) from *s*(*c, q*) proceeds in two steps. First, the full candidate pool is passed through the cross-encoder re-ranking, which scores each chunk against the query, retaining only chunks with a logit score >0. In this way, the cross-encoder also acts as a relevance filter, filtering out chunks with a score of ≤ 0. The surviving candidates, along with their CE scores, are then passed through MMR-based diversification to suppress redundant chunks from the same source document, yielding the final context set:

$$\begin{array}{l} C^{*}\left(q\right)=\text{MMR}\left(Top-k\left\{s_{\text{CE}}^{\prime }\left(c,q\right)\right\},\;\lambda \right) \end{array}$$ (3)

where $s_{\text{CE}}^{\prime }$ represents the Cross-Encoder scores after applying the figureboost equation, and λ∈[0, 1] is the MMR diversity weight balancing query relevance against inter-chunk novelty (empirically set to λ = 0.6). The full details of cross-encoder re-ranking and MMR diversification are provided in Stages 3 and 5 of Section 3.1.2, respectively. By mapping the cross-modal signal as a discrete scalar modification via *f*_boost_ rather than forcing text and visual features into a shared, unified cross-modal embedding space, FFHRAG completely circumvents the representation noise and semantic warping inherent in joint modality projections. This design ensures that the final text chunk selection remains entirely unpolluted by high-dimensional visual vector alignments, while guaranteeing that the generated summary is firmly grounded in the most visually and textually corroborated literature available.

#### 3.1.2 FFHRAG system architecture

The Figure-First Hybrid RAG architecture is designed as a modular, six-stage pipeline that prioritizes visual evidence to guide the selection of textual information. Unlike traditional multimodal systems that require complex cross-modal embedding spaces of textual and visual evidence, FFHRAG employs a figure-first retrieval strategy, where initial discovery of visual content directly influences the scoring of textual candidates provided to the generator. The logical flow of the pipeline is detailed below.

##### 3.1.2.1 Stage 1: Multi-channel figure retrieval

The pipeline begins by identifying relevant visual evidence from the figure corpus using a four-channel retrieval strategy. This multi-modal approach is designed to capture relevant figure signals by leveraging both semantic and lexical features:

- **Channel 1—Dense PubMedBERT captions:** Semantic search over figure-captions using *S-PubMedBERT* embeddings via FAISS index.
- **Channel 2—Dense CLIP captions:** Cross-modal text-image alignment search over figure-captions using a CLIP text encoder via a FAISS index.
- **Channel 3—Dense CLIP image encoding:** Visual similarity search over stored figure images using the CLIP image encoder via a FAISS index.
- **Channel 4—Sparse BM25 captions:** BM25 keyword search over figure-captions to capture exact lexical matches.

The resulting four ranked lists are fused into a unified candidate pool using Reciprocal Rank Fusion (RRF) with constant *k* = 60. We use Reciprocal Rank Fusion here as it provides a robust and parameter-free method for fusing heterogeneous signals from different modalities without requiring complex normalization of raw scores (Cormack et al., 2009). A Cross-Encoder ncbi-MedCPT-Cross-Encoder (Jin et al., 2023a) is subsequently applied across all candidate query-caption pairs to perform joint semantic reranking and relevance filtering. The Cross-Encoder computes fine-grained cross-attention scores across the fused candidate pool; candidates scoring below the relevance threshold (logit ≤ 0) are discarded as values below this threshold indicate that the caption-query pair is irrelevant. All surviving figures are reranked strictly by their Cross-Encoder logits. This ensures that visual assets with unverified textual alignment are discarded prior to final selection. From the CE filtered pool, the top-5 figures are selected, subject to a maximum of 2 figures per source document (PMCID), preventing any single paper from monopolizing the visual signal. The PMCIDs of these selected figures form the Boosted PMCIDs set used in the subsequent stage.

Importantly, the figure and text retrieval pipelines operate independently. In cases where figure retrieval surfaces no candidates above the Cross-Encoder relevance threshold—a logit score of 0, below which all caption-query pairs are considered as irrelevant, the Boosted PMCID set is essentially an empty set (B(*q*) = ∅). Under this condition, the Figureboost mechanism has no effect on the text scoring and the pipeline operates as a text-only retriever. This threshold-based scoring ensures that low-confidence and irrelevant captions do not introduce noise into the retrieval process, preserving answer quality even when figure evidence is unavailable.

##### 3.1.2.2 Stage 2: Hybrid text retrieval

In parallel, a separate retrieval pipeline processes the textual corpus of 1.1 million chunks using a hybrid approach:

- **Dense search:** Embeddings produced from PubMedBERT are used to retrieve the top-100 semantically similar documents using a FAISS index.
- **Sparse search:** A BM25s weighted index is used to select the top-100 keyword-related documents based on the lexical score.

The two ranked lists are fused via RRF (*k* = 60) to produce a consolidated relevance score for each candidate text chunk.

##### 3.1.2.3 Stage 3: Cross-encoder reranking

The consolidated candidate list generated by the RRF sequence is truncated to the top-100 highest-ranked passages and passed directly through a Cross-Encoder (ncbi-MedCPT-Cross-Encoder) to perform fine-grained token-level relevance modeling on each (query, chunk) pair. The cross-encoder outputs a logit relevance score, where values ≤ 0 correspond to irrelevant pairs. Chunks below this threshold are discarded, ensuring only strictly relevant passages proceed to the diversification stage. The resulting raw logit output *s*_CE_(*c, q*) completely replaces the relative, rank-based RRF score, anchoring the primary text retrieval pool in explicit textual alignment. These top-100 prioritized chunks, embedded with their baseline logit representations, are then transmitted directly to the subsequent stage for figure-guided amplification.

##### 3.1.2.4 Stage 4: Figure-guided boosting (Figureboost)

This step constitutes the core novelty of the FFHRAG method, introducing a late-stage, continuous additive adjustment that modulates text-centric baseline scores using upstream visual evidence. The architecture computes a highly calibrated, context-aware boost value based on the semantic alignment between the text chunk and the figures belonging to its parent document. Formally, for any candidate text chunk *c* whose parent document identifier belongs to the boosted PMCID set (B(*q*)), its final combined relevance score $s_{\text{CE}}^{\prime }\left(c,q\right)$ is defined in Equation 4 as:

$$\begin{array}{ll} & s_{\text{CE}}^{\prime }\left(c,q\right)\\ = & s_{\text{CE}}\left(c,q\right)+\beta \cdot \underset{f\in F_{c}}{\max}\left(w_{\text{visual}}\left(f\right)\cdot \max\left(0,e_{c}^{⊤}e_{f}-\tau_{\text{margin}}\right)\right) \end{array}$$ (4)

where *s*_CE_(*c, q*) represents the baseline Cross-Encoder logit score, and β is a global scaling factor governing the maximum allowable cross-modal amplification, empirically set to 2.0. The subset F_*c*_ denotes the collection of top-ranked figures retrieved for query *q* that originate from the exact same source document as chunk *c* that have successfully passed the Cross-Encoder semantic filter. The architecture employs a max-pooling operator ($\underset{f\in F_{c}}{\max}$) over this filtered subset to isolate the single strongest visual-textual bridge, preventing artificial score inflation when a document contains multiple relevant visual assets.

Within this boosting term, *w*_visual_(*f*) represents a normalized confidence weight that scales the boost linearly according to the figure's relative performance. Rather than utilizing a naive rank position, this weight is computed via a min–max normalization over the raw Cross-Encoder logit scores of the surviving figure candidate pool, mapping the highest-performing figure to 1.0 and the lowest to 0.0. The localized cross-modal alignment is calculated as the dot product $e_{c}^{⊤}.e_{f}$. Because the multi-task embeddings are explicitly *L*_2_ normalized during encoding, this dot product yields the exact cosine similarity between the text chunk and the figure caption. To maximize domain-specific retrieval dynamics, these embeddings are generated using specialized asymmetric biomedical dual-encoders, mapping the text chunk (*e*_*c*_) via the MedCPT Article Encoder and the figure caption (*e*_*f*_) via the MedCPT Query Encoder. To guarantee that low-confidence associations do not introduce cross-modal representation noise, a hard similarity margin threshold τ_margin_ (set to 0.80) is subtracted from this alignment. A hinge-loss style rectification operator (max(0, ·)) ensures that only highly specialized, tightly bound semantic relationships exceeding this margin can trigger a positive boost. For all candidate text chunks whose PMCIDs do not appear in the boosted set B(*q*) or those whose scores do not surpass the threshold, the second term drops to zero, and they safely retain their original baseline Cross-Encoder score.

##### 3.1.2.5 Stage 5: Context diversification via MMR

To prevent redundant passages from the same source document dominating the generator's context window, Maximal Marginal Relevance (MMR) (Carbonell and Goldstein, 1998) is applied to the top-50 chunks that surpass the cross-encoder thresholding. To balance computational efficiency with high-fidelity relevance scoring, we implement an asymmetric MMR function. For a candidate chunk *c* in the unselected pool *C*, where *S* is the set of already selected chunks and *q* is the query, Equation 5 describes score computed as:

$$\begin{array}{l} \text{MMR}\left(c\right)=\lambda \cdot s_{\text{CE}}^{\prime }\text{norm}\left(c,q\right)-\left(1-\lambda \right)\underset{s\in S}{\max}{\text{Sim}}_{\text{Bi}}\left(c,s\right) \end{array}$$ (5)

where CE_norm_(*c, q*) represents the min-max normalized cross-encoder logit acting as the strict relevance metric, and Sim_Bi_(*c, s*) represents the bi-encoder cosine similarity penalty applied against previously selected chunks. When the value of λ = 1, the system computes a standard relevance-ranked list, and when λ = 0, it computes maximal diversity ranking (Carbonell and Goldstein, 1998). We use a diversity factor of λ = 0.6, ensuring that MMR iteratively selects chunks that balance query relevance with inter-chunk novelty, yielding a final set of *k* = 20 diverse context passages. As far as retrieval evaluation goes, the entire set of 20 passages is used; however, the selection is further refined to include a smaller set of text blocks within the generation prompt to maintain informativeness while minimizing the risk of distracting the model from its task. The number of chunks injected per-variant is discussed in 3.2.6.

##### 3.1.2.6 Stage 6: Grounded generation

The final context, which combines this small set of text chunks and captions of the top-3 images ranked according to their Cross-Encoder logit, is formulated in the generator prompt. It is important to mention that despite using all five retrieved images to calculate evaluation measures (figure precision, figure recall, and figure hit rate), only three top-ranked images are included within the generation prompt.

The complete architecture is illustrated in Figure 1.

**Figure 1:**
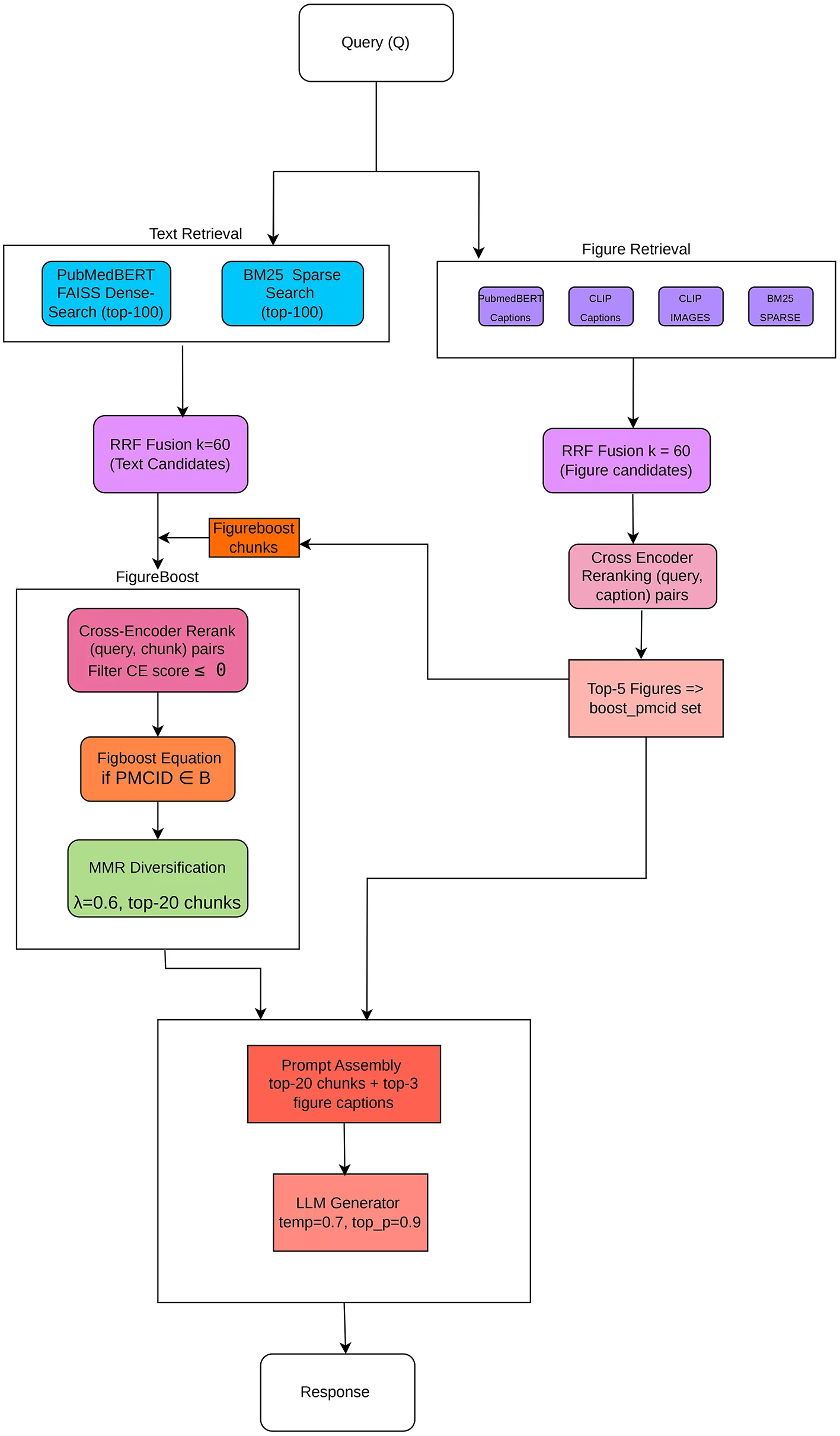
Figure-First Hybrid RAG architecture.

### 3.2 Experimental setup

This section defines the technical setup and the implementation details required to evaluate the FFHRAG pipeline. We detail the construction of our large-scale biomedical corpus, the characteristics of our evaluation dataset, and the specific configurations used for data preprocessing, indexing, and generator prompting.

#### 3.2.1 Corpus and dataset characterization

To maintain a clear distinction between our retrieval source and our testing framework, we categorize our data into *Knowledge Corpus*, the retrieval source, and *Evaluation Dataset*, the dataset used for evaluating our framework.

##### 3.2.1.1 Knowledge corpus

The retrieval corpus is constructed from a curated subset of the PubMed Central Open Access (PMC-OA) collection, accessed via the PMC OA API (National Library of Medicine, 2003). We retrieved a total of **29,532** papers, among which **20,295** contain full TGZ archives containing complete manuscript text and figure assets while the remaining **9,237** papers contribute abstract-level text only. The abstract-only subset arises predominantly from source papers referenced in the BioASQ-13b task B ideal-answer generation benchmark, where full-text archives are not publicly available via the PMC OA API. The distribution of the domain of the corpus is designed to mirror the broader distribution of the PMC OA corpus. We downloaded papers, spanning the 22 standard domains in biomedical research, which serves to rigorously test the system's cross-domain generalization. The largest concentrations are in the Genetics/Genomics & Molecular Biology (9,470 papers), and Oncology & Neoplasms (4,900 papers) domains. Domains which contained papers less than 1.5% of the total corpus were classified as Other Specialized Disciplines. These include Pulmonology, Psychiatry and psychology, Rheumatology and autoimmune, Urology, Orthopedics and musculoskeletal, Hematology, Ophthalmology and vision, Nephrology, Dermatology, and Geriatrics and aging. The distribution of the domains is illustrated in Figure 2.

**Figure 2:**
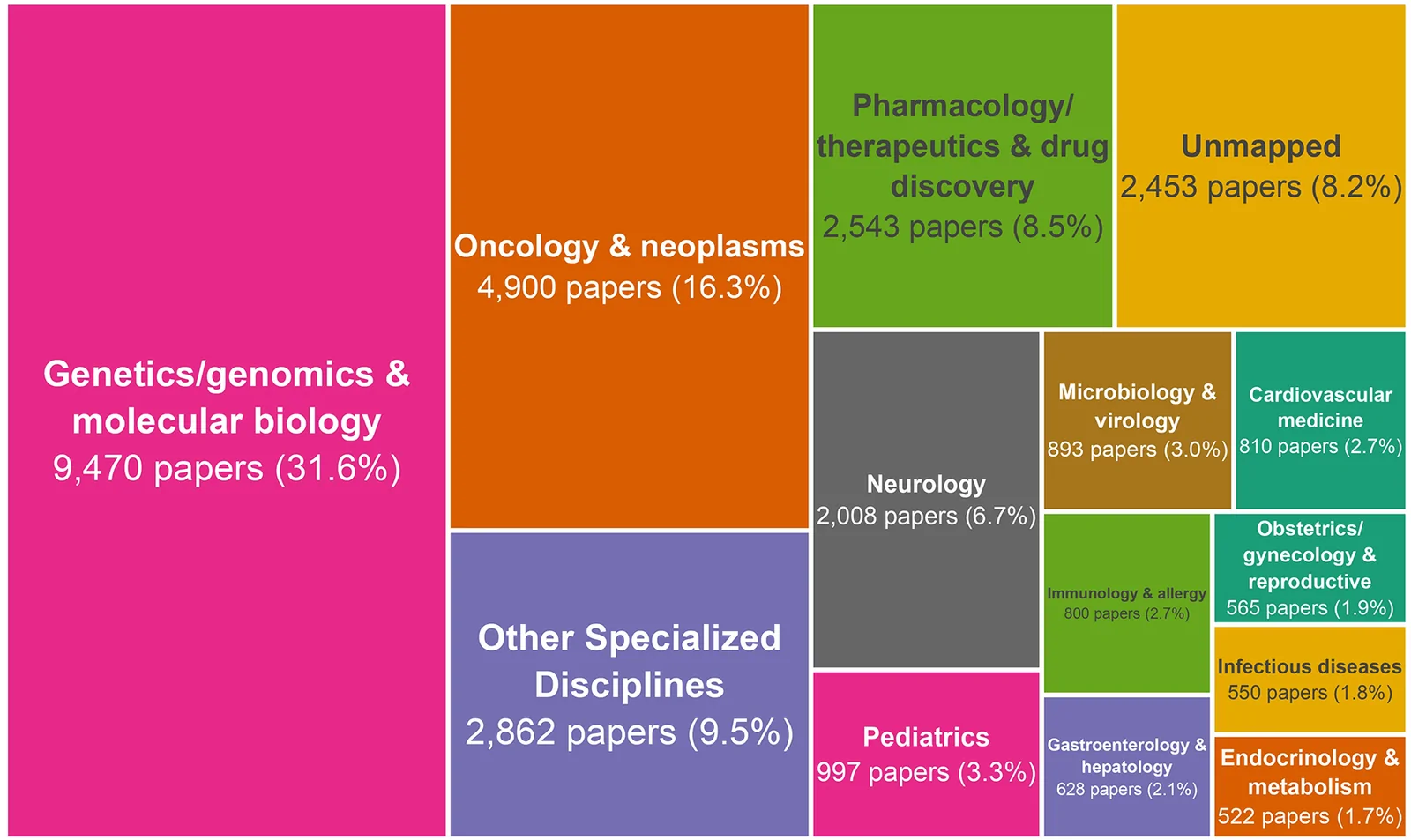
Domain-distribution of the constructed corpus.

##### 3.2.1.2 Evaluation dataset

We utilize a specialized subset of the BioASQ-13b task B challenge (Nentidis et al., 2026), filtering specifically for *summary*-type questions that require paragraph-length synthesis rather than factoid extraction. The full BioASQ-13b summary split contains over 1,200 such questions, from which we derive two purpose-built evaluation subsets corresponding to the two phases of our empirical study.

**Phase 1—Generation quality evaluation:** In order to identify the best-performing generator model for biomedical query summarization, we created a subset of **650 questions** from the full BioASQ 13-b summary question set, retaining only those questions whose *expert-provided* answers are around 70–100 words. This length constraint ensures that reference answers reflect the expected output format for biomedical query summarization, providing a fair and consistent basis for comparing generation quality across multiple LLMs.

**Phase 2—Retrieval evaluation:** In order to comprehensively evaluate the retrieval pipeline, we created a set of 290-summary type questions. Each query in this set was enriched with gold-standard relevant document IDs (PMCIDs), derived from BioASQ expert annotations. To analyze the specific impact of the figure-first signal, we stratify this set into two categories based on the inherent visual necessity of the query:

- **Figure-True (84 questions, 28.9%):** These queries target complex biological processes or structural relationships where visual evidence is essential for a complete clinical understanding.
- **Figure-False (206 questions, 71.1%):** These consist of broader, descriptive queries that are traditionally resolved through high-level textual summaries and do not strictly require visual supplements.

This stratification serves a dual empirical purpose. First, it allows us to qualify the performance differential between figure-dependent and text-centric subsets. Second, it allows us to evaluate the robustness of the pipeline against injection noise, specifically, whether providing figure-captions as context for questions that ideally do not require figures negatively impact the generator's performance.

To ensure a rigorous benchmark, all 290 questions underwent a manual alignment process, conducted entirely prior to and independent of any retrieval system run, eliminating the possibility of annotation bias from system output. For each question, relevant text chunks were identified by locating source documents within our PMC-OA corpus, whose content directly addressed the query's information need. For the 84 Figure-True questions, figure gold labels were assigned by independently searching the corpus for figures whose caption or visual content directly provided supplementary evidence central to the query, without reference to which PMCIDs appear in the text gold set. Each question which requires figures were annotated with 3–5 figures. This process ensures that figure and text gold annotations are semantically independent, and that our metrics accurately capture the system's ability to navigate the specific 1.1 million text chunks and 82,369 figure-captions used in this study.

##### 3.2.1.3 Figure relevance metrics and annotation guidelines

Figure relevance was adjudicated strictly based on additive evidential utility. A figure was incorporated into the gold-standard context set if and only if its cross-modal components—either the visual graphic itself or its explicit caption—provided substantive, non-redundant grounding that extended beyond the scope of the corresponding textual annotations. Specifically, candidate figures were required to explicitly depict a biological mechanism, an empirical experimental outcome, or a spatial-structural relationship that measurably fortified the factual matrix available to the downstream generator for a given query. To operationalize this standard during manual curation, all co-indexed figures were processed through a strict binary classification framework (Relevant vs. Irrelevant), ensuring absolute distinction between superficial visual decoration and core evidentiary assets.

To ground this abstract relevance framework in concrete experimental terms, we present three representative evaluation examples. These cases explicitly demonstrate the operational boundary between non-redundant multimodal grounding and purely decorative visual duplication.

1. **Example 1 [Fig-True: mechanistic pathway dependency]**
  - *Query:* “What is the function of the Spt6 gene in yeast?”
  - *Gold Text Answer Matrix: Spt6 is a highly conserved histone chaperone that interacts directly with RNA polymerase II and histones to regulate gene expression. It is essential for transcription elongation, chromatin remodeling, and the global repression of intragenic cryptic initiation and antisense transcription*.
  - *Figure 3.1* *Representation (Epigenetic and Phenotypic Grounding taken from PMCID 6125631 (Gouot et al.*, *2018**)):*
    - *Visual contents:* Multi-panel layout containing phenotypic yeast spot assays, Northern blot bands for the *FLO8* locus, and Chromatin Immunoprecipitation (ChIP) bar charts tracking histone occupancy.
    - *Core Caption Highlight:* Shows that mutating the CK2 phosphorylation sites in Spt6 (*spt*6-7SA) induces a thermosensitive phenotype, causes a failure to suppress spurious transcription from the *FLO8* cryptic promoter, and significantly increases replication-independent H3K56ac acetylation levels across coding regions.
  - *Figure 3.2* *Representation [High-Density Genomic and Transcriptomic Tracks taken from PMCID 6125631 (Gouot et al.*, *2018**)]:*
    - *Visual contents:* Global RNA-seq metagene profile graphs aligned across Transcription Start Sites (TSS) and Transcription End Sites (TES), scatter-plot correlations, multi-track genomic line profiles for the *PHO84* locus, and Venn diagrams.
    - *Core caption highlight:* Demonstrates the global role of Spt6 phosphorylation in repressing antisense transcription. The visual track profiles explicitly map sense vs. antisense RNA-seq signals at the *PHO84* locus in WT vs. spt6-7SA. Genome-wide Venn diagrams and correlation plots further confirm a significant spatial overlap in antisense enrichment between *ck*2^*ts*^ and *spt*6-7SA mutants.
  - *Reasoning for Fig-True classification:* While the text-only reference provides a high-level overview of Spt6's function, it merely presents the final conclusion without providing the evidence necessary for its substantiation. In molecular genetics, one typically demonstrates how a gene normally functions by obtaining a mutant, or a disabled form and making observations on what goes wrong in the system. Figures 3.1 and 3.2 provide the experimental evidence required to substantiate the conclusion. The two figures demonstrate how *spt*6-7SA mutant is capable of inducing improper cryptic and antisense transcription in certain loci of genome (such as *FLO8* and *PHO84*), thus providing the crucial data for specifying the regulatory function of the wild-type gene. This visual documentation represents the irreplaceable evidence that bridges the gap between a flat text assertion and verifiable biological reality. As this experimental backing cannot be replicated by linear text chunks, the figure assets provide genuine utility to the query, and the query therefore classified as figure-dependent (**Fig-True**).
2. **Example 2 [Fig-True: direct mechanistic and pathological grounding]**
  - *Query:* “What is the role of AMPK in diabetic cardiomyopathy?”
  - *Gold Text Answer Matrix: AMPK activation protects cardiac structure and function by restoring cardiac autophagy and protecting against apoptosis in the diabetic heart. Mechanistically, this is driven by the dissociation of Bcl-2 from Beclin1. Conversely, genetic inhibition of AMPK suppresses autophagy and exacerbates cardiac dysfunction, while pharmacological stimulation via metformin rescues this phenotype*.
  - *Figure 4.1* *Representation (Molecular Mechanism and Autophagy Assays) taken from PMCID 3609561 (He et al.*, *2013**):*
    - *Visual contents:* Multi-panel layout featuring Western blot bands and densitometric bar charts tracking protein phosphorylation (AMPK, ACC, and JNK1), co-immunoprecipitation (IP) assays for the Bcl-2–Beclin1 complex, and quantitative LC3-II autophagy expression tracks.
    - *Core Caption Highlight:* Demonstrates that AMPK activation physically disrupts the repressive Bcl-2–Beclin1 association to successfully restore cellular autophagy in diabetic mouse models, validating these changes across both genetic mutant (DN-AMPKα2) and metformin-treated groups.
  - *Figure 4.2* *Representation (Downstream Pathological Tissue Outcomes) taken from PMCID 3609561 (He et al.*, *2013**):*
    - *Visual Contents:* Histological panels displaying fluorescent TUNEL staining of apoptotic heart tissue sections alongside Western blot evaluations of downstream apoptotic executioner proteins (cleaved caspase-3 and cleaved PARP).
    - *Core Caption Highlight:* Quantifies tissue-level cell death rates, proving that AMPK activation directly suppresses cardiac apoptosis and limits cellular drop-out under diabetic stress conditions.
  - *Reasoning for Fig-True classification:* Although the text reference offers an exhaustive map of the signaling pathways involved, it renders these complex biological relationships as simplistic statements without any real-world application. Figures 4.1 and 4.2 offer the necessary experimental proof needed to fill the gap created by such an approach. Every statement made in the text is backed up directly by an experiment featured in the figure—the text mentions chemical dissociation, which is proved through the use of binding assays in Figure 4.1, and protection against cell death, which is proved through the process of tissue staining depicted in Figure 4.2. Through the presentation of these figures, the molecular switches can be demonstrated physically to verify the protective power of the gene, making the asset figure-dependent (**Fig-True**).
3. **Example 3 [Fig-False: declarative definition and symptom lookup]**
  - *Query:* “What is the “flight-or-fight response?”
  - *Gold Text Answer Matrix: This is also known as the “flight-or-fight response” and is defined as an individual's response to a stimulus such as stress, that includes loss of sleep, anxiety, shortness of breath, muscle and joint pain*.
  - *Reasoning for Fig-No Classification:* This query epitomizes the typical form of descriptive queries, targeting the classic definition of a psychological term and its check-list of symptoms. The text-only gold answer resolves the query satisfactorily, because it identifies the criteria for diagnosis (anxiety, insomnia, dyspnea and arthralgia). As opposed to the above types of molecular queries, no spatial reasoning or three-dimensional geometry is needed to tackle this question. Any figure included in the analysis of this type of query is used solely for illustrative purposes and will offer witnesses no new empirical evidence beyond the one found in the text itself. Therefore, it is classified as strictly figure-independent (Fig-False).

**Figure 3:**
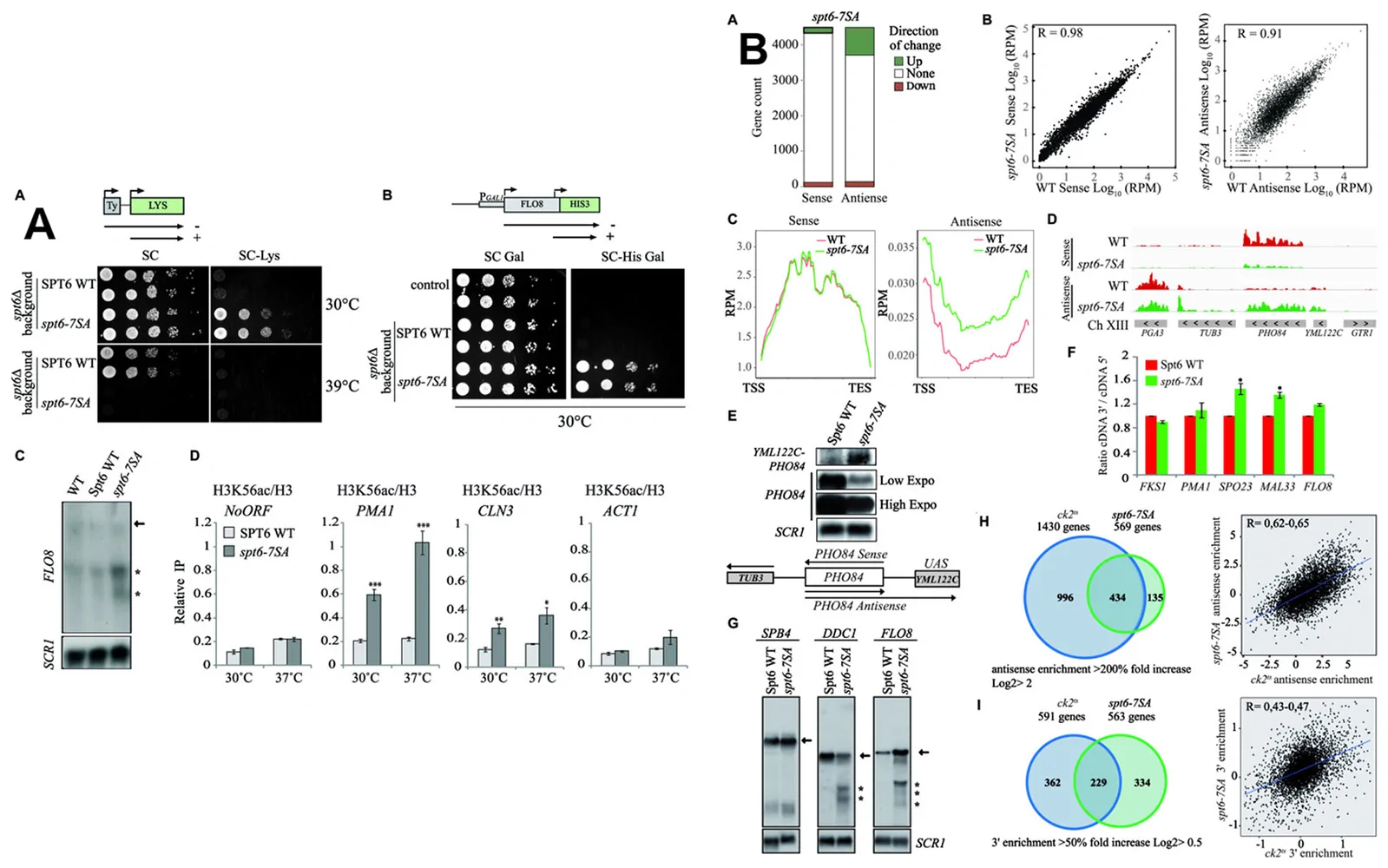
Empirical verification and multimodal grounding for the regulatory function of the yeast Spt6 gene under wild-type and *spt*6-7SA mutant conditions. **(A)** Panels A–D: Epigenetic and phenotypic analysis tracking yeast spot assays, Northern blot bands (*FLO8*), and quantitative ChIP H3K56ac acetylation levels. **(B)** Panels A–I: High-density global RNA-seq metagene profiles aligned across genomic coordinates and cross-locus track alignments (*PHO84*). Reproduced from (Gouot et al., 2018) under the Creative Commons Attribution-NonCommerical (CC BY-NC 4.0). Original internal panel lettering is preserved from the source publication.

**Figure 4:**
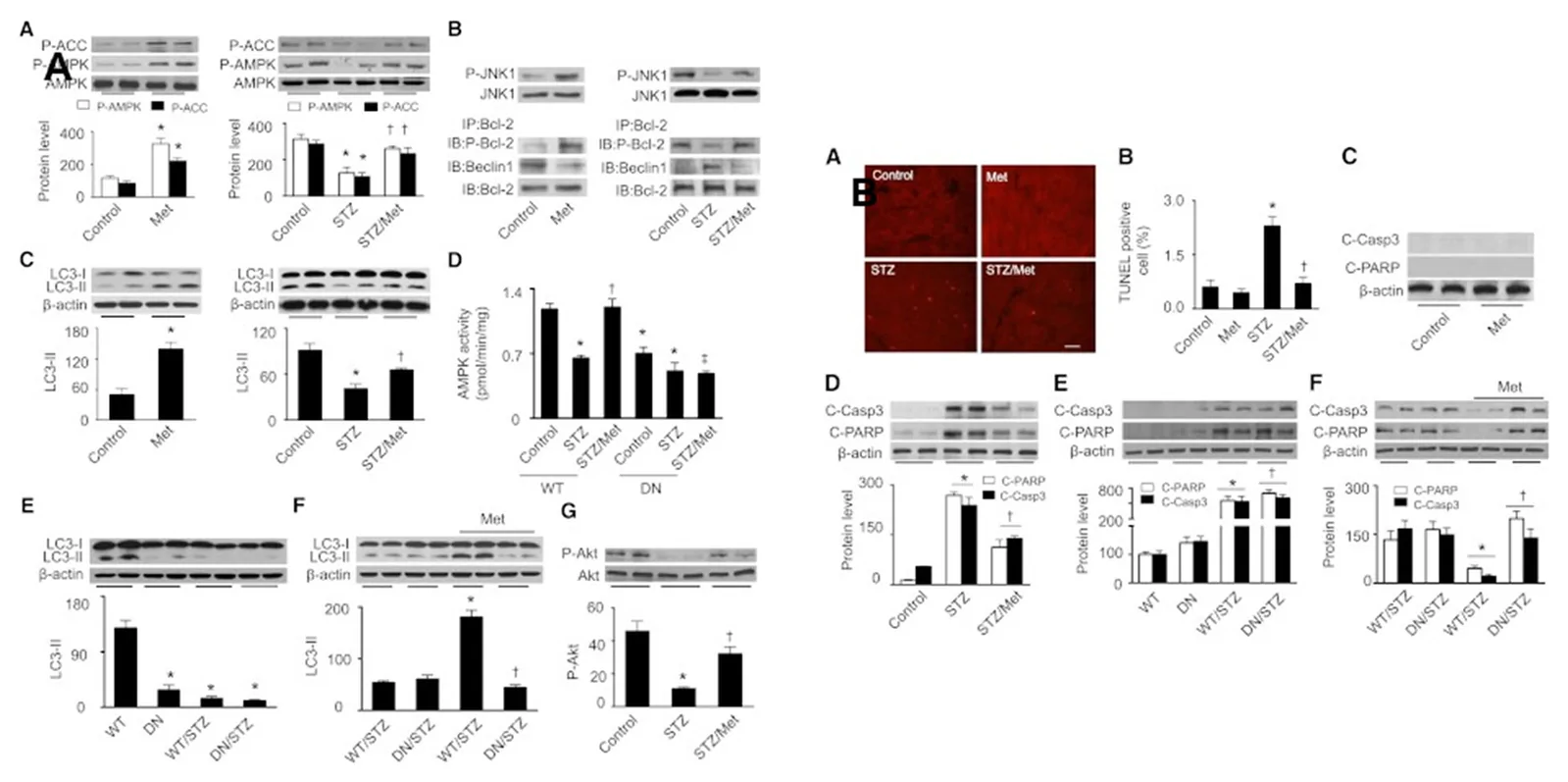
Mechanistic signaling and corresponding histopathological validation tracking the protective role of AMPK activation against diabetic cardiomyopathy parameters. **(A)** Panels A–G: Biochemical profiling featuring Western blots and co-immunoprecipitation (IP) of the target Bcl-2–Beclin1 dissociation complex alongside LC3-II markers. **(B)** Panels A–F: Downstream cellular pathology mapping fluorescent tissue-level TUNEL apoptosis staining and cleaved caspase-3/PARP markers. Reproduced from (He et al., 2013) under the Creative Commons Attribution-NonCommercial-NoDerivatives (CC BY-NC-ND 3.0) license. Original internal panel lettering is preserved from the source publication.

Due to the highly specialized biomedical nature of the queries, dataset curation followed a multi-tiered Expert Adjudication and Consensus Framework rather than a traditional crowd-sourced model. The primary author established baseline annotations under a strict structural rubric, which were subsequently audited and refined through collaborative cross-examination with two senior faculty co-authors. All ambiguous edge cases were iteratively resolved to achieve 100% expert consensus. Because the final gold-standard dataset represents a fully reconciled collective agreement rather than parallel, independent observations, calculating traditional inter-annotator reliability metrics—such as Cohen's *k* was rendered mathematically inapplicable.

#### 3.2.2 Data preprocessing

##### 3.2.2.1 Text extraction and chunking

Full-text papers are distributed by PMC-OA in NXML format, a structured XML schema conforming to the JATS publishing standard. We use Python's xml.etree.ElementTree library to parse each NXML file. We go through the  < body> element recursively to get all the  < sec> nodes. For each section record, it saves the section ID, the parent section ID, the hierarchical section path, the title, and the combined text of all the  < p> paragraph elements in that section. These are saved to sections.jsonl, with each line representing one section and all records linked to their source paper by PMCID. After that, each section is split into text chunks that are no longer than 1,500 characters. This creates a file called chunks.jsonl, where each chunk record has its own chunk_id (in the format PMCID__sec_id__cXXXX), PMCID, section identifier, character offsets, and the text of the chunk. This dual-file design enables *hierarchical expansion* during retrieval: the system initially retrieves at the chunk level for precision, and optionally expands a retrieved chunk to its full parent section for broader context.

Abstract-only papers, which lack an NXML body structure, are processed separately by treating the abstract text as a single flat section and applying the same 1,500-character chunking window. This ensures all 29,532 papers contribute to the retrieval corpus, with abstract-derived chunks participating in both dense and sparse retrieval while naturally being excluded from figure-based boosting due to the absence of associated figure assets. Across the 29,532 full-text and abstract-alone papers, this process yields a total of **1,101,618 text chunks** spanning the 22 biomedical domains.

##### 3.2.2.2 Figure extraction

Figure assets and their associated captions are extracted in parallel from the same NXML files. Each figure record stores the PMCID, figure identifier, caption text, and the path to the corresponding image file where available. All figure records are linked to their source documents via PMCID, enabling the Figureboost mechanism in Stage 4 of 3.1.2 to resolve cross-modal signals back to their originating papers. The final figure corpus consists of **82,369 figure-caption records**.

#### 3.2.3 Embedding generation

**Text and figure-caption embeddings(PubMedBERT)** All text chunks and figure-captions are encoded using pritamdeka/S-PubMedBert-MS-MARCO (Deka et al., 2022), a domain-adapted variant of PubMedBERT fine-tuned on the MS-MARCO passage retrieval task via the Sentence-BERT framework. This model produces 768-dimensional dense vector representations that capture nuanced biomedical semantic relationships, making it well-suited for both chunk-level retrieval and caption-level figure matching. Embeddings are computed in batches of 128 on GPU and are L2-normalized prior to storage. This normalization ensures that inner product search over the resulting FAISS index is mathematically equivalent to cosine similarity, providing a robust measure of semantic alignment. The process yields **1,101,618 chunk embeddings** and **82,369 figure-caption embeddings**, each of dimension 768, stored as float32 NumPy arrays.

**Image and figure-caption embeddings (BiomedCLIP)** For multimodal figure retrieval, we employ BiomedCLIP-PubMedBERT_256-vit_base_patch16_224 (Zhang et al., 2024), a vision-language model pre-trained on biomedical image-caption pairs from PubMed Central. BiomedCLIP provides a shared 512-dimensional embedding space for both images and text, enabling direct cross-modal similarity comparison without requiring a separate alignment step.

Two distinct embedding sets are generated from this model. The **image embeddings** are produced by passing each figure image through BiomedCLIP's ViT-B/16 image encoder, preceded by the model's native preprocessing transform. The **caption embeddings** are produced by encoding each figure's caption text through BiomedCLIP's text encoder, with captions truncated to a maximum of 256 tokens. Consistent with our text retrieval setup, all BiomedCLIP embedding vectors are L2-normalized prior to storage, yielding 82,369 vectors of dimension 512 per modality stored as float32 NumPy arrays. This design supports the three figure retrieval channels in Stage 1 of the FFHRAG pipeline: dense semantic search over captions via PubMedBERT (768-dim), cross-modal text-image search via CLIP text encoder (512-dim), and visual similarity search via CLIP image encoder (512-dim).

#### 3.2.4 FAISS index construction

We construct four distinct FAISS indices over the embedding sets described in Section 3.2.3, each supporting inner product search over L2-normalized vectors to achieve a mathematically equivalent result to cosine similarity. For large-scale indices where *n*≥10, 000, we utilize an IVFFlat index (Inverted File with Flat quantization) to ensure sub-linear search time (Johnson et al., 2021). For these indices, the number of Voronoi cells is defined as $n_{\text{list}}=\min\left(\left\lfloor \sqrt{n}\right\rfloor ,4,096\right)$, and we set *n*_probe_ = 64 during query time to balance retrieval latency against recall. For indices containing fewer than 10, 000 records, we employ an exact IndexFlatIP search.

The characteristics of the four indices are detailed below:

- **Text Chunk Index** (text_chunks_index.faiss): Indexes all **1,101,618** text chunks derived from full-text and abstract-only papers, encoded with S-PubMedBERT-MS-MARCO (768-dim). To manage memory constraints during construction, embeddings from disparate sources (Full-text and abstract-only embeddings) are concatenated into a single contiguous array using memory-mapped loading before index training.
- **figure-caption BERT Index** (figure_captions_bert_index.faiss): Indexes **82,369** figure-captions encoded with S-PubMedBERT-MS-MARCO (768-dim). This index supports the high-precision semantic caption retrieval defined in Channel 1 of the FFHRAG pipeline.
- **figure-caption CLIP Index** (figure_captions_index.faiss): Indexes **82,369** figure-captions encoded with the BiomedCLIP text encoder (512-dim), supporting the cross-modal text-image retrieval used in Channel 2.
- **Figure Image CLIP Index** (figure_images_index.faiss): Indexes **82,369** figure images encoded with the BiomedCLIP ViT-B/16 image encoder (512-dim), enabling visual similarity search in Channel 3.

Each index is persisted with a corresponding .npy ID array mapping FAISS row positions to their respective chunk_id or PMCID_fig_id identifiers. This allows for rapid metadata resolution at query time without the overhead of loading the full metadata corpus into memory.

#### 3.2.5 BM25 sparse indexing

To complement the dense semantic retrieval, we construct two sparse retrieval indices designed to capture exact lexical matches that the embedding-based models might overlook, particularly for rare clinical terminology and gene identifiers.

**Text Sparse Indices (BM25+)** For textual evidence retrieval, we implement a BM25 sparse index over the 1,101,618 chunk corpus.

- **BM25+ Index:** We built this with the bm25s library, which uses the BM25+ variant to give better scores to low-frequency terms without punishing long documents too much. Before indexing, texts are broken up into tokens and English stopwords are removed (Robertson and Zaragoza, 2009).

The resulting sparse matrix is stored in CSR format for efficient query-time scoring.

**Figure-Caption BM25 Index** A dedicated **BM25+** index is constructed over the 82,369 figure-captions. This index serves as Channel 4 of the Stage 1 figure discovery process, ensuring that keyword-level query-caption matches are captured for specific visual evidence that may be under-weighted by dense BERT or CLIP channels. Figure identifiers are mapped via a parallel bm25_figures_ids.pkl array to enable direct linkage to figure metadata during Stage 1 discovery.

#### 3.2.6 Generation prompt design and post-processing

To evaluate the incremental contributions of our architectural components, we utilize a variant-aware prompt builder that dynamically adjusts context density based on the specific ablation variant being tested. These variants, which form the basis for our Phase-2 retrieval and generation analysis (refer Section 4), have different limits on the length of the context and the number of document units.

**Variant-aware context configuration** We define six ablation variants: Vanilla, BM25, CE Filter, Hierarchical, FigureBoost, and the complete FFHRAG pipeline. As summarized in Table 1, richer variants come with larger context windows (1,500 characters per passage) so that the generator can use the deeper evidence it gets from hierarchical expansion and figure-guided boosting. On the other hand, baseline variants (Vanilla, BM25) use a limited window of 700 characters to mimic standard RAG limits.

**Table 1:** Prompt configuration per ablation variant.

Variant	Unit	Count (*k*)	Char limit
Vanilla/BM25	Chunk	10	700
CE Rerank	Chunk	8	1,500
Hierarchical	Section	4	2,500
FigureBoost	Chunk	8	1,500
FFHRAG	Chunk	8	1,500

**Prompt construction:** The prompt is assembled into a three-tier structure. The complete prompt is described in depth in Table 2:

- **Figure context block:** This block is exclusive to the FFHRAG variant. This block prepends the top-3 figure-captions (truncated to 180 characters) as [Figure i]: < caption>. This provides the primary visual evidence for the LLM.
- **Text evidence block:** This block contains *k* retrieved passages formatted as [Passage i]: < text>. For the Hierarchical variant, the chunks are expanded into full parent sections, whereas for the other variants, only chunks are injected.
- **Instruction block:** Instructions defining the “Biomedical Expert” persona and enforcing strict constraints: 60–80 word target length, English-only response, and a total prohibition of internal citations or passage markers to ensure evaluation against the ideal answers provided in the BioASQ dataset.

**Table 2:** The structured, variant-aware prompt template used for the FFHRAG pipeline.

**System instructions:** You are a biomedical expert. Answer the question using the provided evidence only.

**# Question**
{query}

# Evidence
[Figure i]: < caption_text>…
[Passage i]: < text_evidence>…

**# Instructions**
•Write 3–4 clear sentences (60–80 words). •Include key mechanisms, genes, or pathways mentioned. •Use precise biomedical terminology. •Answer in English only. •Do **NOT** include citations, references, or passage numbers.

For ablation variants, the figure context block is omitted, and the text passage limit is adjusted.

**Response refinement**

Post-generation, answers undergo refinement via a clean_answer function. This stage utilizes regular expressions to strip residual reference markers (e.g., [1], [Passage n]) and model preamble artifacts (e.g., “Based on the provided evidence…”). To maintain parity across variants during evaluation, a truncate_to_target mechanism is employed; any response exceeding 100 words is trimmed to the nearest sentence boundary, preserving semantic completeness while enforcing the word-count consistency required for fair ROUGE and Bert-Score comparisons.

#### 3.2.7 Evaluation metrics

To systematically assess both the generator selection and the retrieval pipeline, evaluation metrics are organized into two phases corresponding to the empirical design described in Section 2.

##### 3.2.7.1 Phase 1—Generation quality metrics

Phase 1 evaluates the quality of biomedical summaries produced by six candidate LLMs against BioASQ gold ideal answers. The following metrics are used:

- **ROUGE-1 and ROUGE-L**: Measure unigram overlap and longest common subsequence overlap between the generated answer and the gold reference, respectively (Lin, 2004). To ensure robust evaluation, both metrics are reported as *F*_1_-measures that balance precision and recall. ROUGE-1 is computed as the harmonic mean of unigram recall (*R*_1_) and precision (*P*_1_) Equations 6 and 7 describe these as:
  $$\begin{array}{lll} R_{1} & = & \frac{\sum_{w\in \text{Ref}}\min\left(C_{\text{gen}}\left(w\right),C_{\text{ref}}\left(w\right)\right)}{\sum_{w\in \text{Ref}}C_{\text{ref}}\left(w\right)}\\ P_{1} & = & \frac{\sum_{w\in \text{Ref}}\min\left(C_{\text{gen}}\left(w\right),C_{\text{ref}}\left(w\right)\right)}{\sum_{w\in \text{Gen}}C_{\text{gen}}\left(w\right)}\\ \text{ROUGE-1} & = & \frac{2\cdot R_{1}\cdot P_{1}}{R_{1}+P_{1}} \end{array}$$ (6)
  where *C*_gen_(*w*) and *C*_ref_(*w*) denote the count of unigram *w* in the generated and reference answers respectively. ROUGE-L replaces unigram token matching with the Longest Common Subsequence (LCS), similarly evaluated as an *F*_1_-measure between LCS-based recall (*R*_lcs_) and precision (*P*_lcs_):
  $$\begin{array}{lll} R_{lcs} & = & \frac{\text{LCS}\left(\text{gen},\text{ref}\right)}{\left|\text{ref}\right|},\;P_{lcs}=\frac{\text{LCS}\left(\text{gen},\text{ref}\right)}{\left|\text{gen}\right|},\\ \text{ROUGE-L} & = & \frac{\left(1+\beta^{2}\right)\cdot R_{lcs}\cdot P_{lcs}}{R_{lcs}+\beta^{2}\cdot P_{lcs}} \end{array}$$ (7)
  where β = 1 assigns equal weight to precision and recall, consistent with standard summarization evaluation practice.
- **BLEU**: Measures n-gram precision of the generated answer against the reference with a brevity penalty *BP* (Papineni et al., 2002) as shown in Equations 8 and 9:
  $$\begin{array}{l} \text{BLEU}=BP\cdot \exp\left(\overset{N}{\underset{n=1}{\sum }}w_{n}\log p_{n}\right) \end{array}$$ (8)
  where *p*_*n*_ is the modified n-gram precision for n-grams of order *n*, $w_{n}=\frac{1}{N}$ are uniform weights, and:
  $$\begin{array}{l} BP=\{\begin{array}{cc} 1 & \text{if}|\text{gen}|>|\text{ref}|\\ e^{1-|\text{ref}|/|\text{gen}|} & \text{otherwise} \end{array} \end{array}$$ (9)
- **GLEU**: A sentence-level metric introduced for the Google Neural Machine Translation system (Wu et al., 2016). Unlike BLEU, which measures only n-gram precision, GLEU computes the minimum of n-gram precision and recall across all n-gram orders *n*∈{1, …, *N*}, penalizing both under-generation and over-generation as described in Equation 10:
  $$\begin{array}{ll} \text{GLEU}\left(H,r\right) & \\ & =\min(\frac{\sum_{n=1}^{N}\sum_{g\in {\text{ng}}_{n}\left(H\right)}\min\left(C_{H}\left(g\right),C_{r}\left(g\right)\right)}{\sum_{n=1}^{N}|{\text{ng}}_{n}\left(H\right)|},\\ & \times \frac{\sum_{n=1}^{N}\sum_{g\in {\text{ng}}_{n}\left(H\right)}\min\left(C_{H}\left(g\right),C_{r}\left(g\right)\right)}{\sum_{n=1}^{N}|{\text{ng}}_{n}\left(r\right)|}) \end{array}$$ (10)
  where ng_*n*_(*H*) and ng_*n*_(*r*) denote the set of *n*-grams of order *n* in the generated hypothesis *H* and gold reference *r*, respectively, while *C*_*H*_(*g*) and *C*_*r*_(*g*) represent the frequency counts of *n*-gram *g* within each sequence. By taking the minimum across both dimensions, GLEU provides a balanced evaluation more highly correlated with human judgment at the sentence level than standard BLEU.
- **METEOR**: Evaluates translation quality by computing the harmonic mean of unigram precision and recall, incorporating a penalty for differences in word order (Lavie and Agarwal, 2007) as described in Equation 11:
  $$\begin{array}{l} \text{METEOR}=F_{\text{mean}}\cdot \left(1-\text{Pen}\right) \end{array}$$ (11)
  where *F*_mean_ is the harmonic mean of unigram precision and recall, and Pen is a fragmentation penalty.
- **Bert-Score F1**: Computes token-level semantic similarity using contextual embeddings (Zhang et al., 2020). We use the model *pritamdeka/S-PubMedBert-MS-MARCO*. For generated tokens ŷ and reference tokens *y*, precision *P*, recall *R*, F1 are described in Equation 12 as:
  $$\begin{array}{l} P_{\text{BERT}}=\frac{1}{\left|ŷ\right|}\underset{{ŷ}_{i}\in ŷ}{\sum }\underset{y_{j}\in y}{\max}x_{{ŷ}_{i}}^{⊤}x_{y_{j}},\\ R_{\text{BERT}}=\frac{1}{\left|y\right|}\underset{y_{j}\in y}{\sum }\underset{{ŷ}_{i}\in ŷ}{\max}x_{{ŷ}_{i}}^{⊤}x_{y_{j}}, \end{array}$$
  $$\begin{array}{l} F1_{\text{BERT}}=\frac{2\cdot P_{\text{BERT}}\cdot R_{\text{BERT}}}{P_{\text{BERT}}+R_{\text{BERT}}} \end{array}$$ (12)
  where **x**_*i*_ denotes the contextual embedding of token *i*.
- **SBERT cosine similarity**: Measures holistic semantic alignment between sentence-level embeddings of the generated answer **e**_gen_ and reference **e**_ref_ (Reimers and Gurevych, 2019) this is described in Equation 13 as:
  $$\begin{array}{l} {\text{SBERT}}_{\cos}=\frac{e_{\text{gen}}\cdot e_{\text{ref}}}{\left\|e_{\text{gen}}\|\cdot \|e_{\text{ref}}\right\|} \end{array}$$ (13)
  Embeddings are computed using S-PubMedBERT-MS-MARCO, providing a domain-aware semantic similarity signal robust to paraphrasing.

##### 3.2.7.2 Phase 2—Retrieval and end-to-end metrics

Phase 2 evaluates the full FFHRAG pipeline and its ablation variants across retrieval, passage, domain, figure, and generation dimensions. All Phase 1 generation metrics are retained. The following retrieval-centric metrics are additionally introduced:

- **Precision@5 (P@5)**: The fraction of the top-5 retrieved documents that are relevant as described in Equation 14:
  $$\begin{array}{l} P@5=\frac{\left|\left\{\text{relevant docs}\right\}\cap \left\{\text{top-5 retrieved}\right\}\right|}{5} \end{array}$$ (14)
- **Recall@10 (R@10)**: The fraction of all relevant documents recovered within the top-10 results as described in Equation 15:
  $$\begin{array}{l} R@10=\frac{\left|\left\{\text{relevant docs}\right\}\cap \left\{\text{top-10 retrieved}\right\}\right|}{\left|\left\{\text{relevant docs}\right\}\right|} \end{array}$$ (15)
- **Mean Reciprocal Rank (MRR)**: The mean of the reciprocal rank of the first relevant document across all |*Q*| queries described in Equation 16 as:
  $$\begin{array}{l} \text{MRR}=\frac{1}{\left|Q\right|}\overset{\left|Q\right|}{\underset{i=1}{\sum }}\frac{1}{{\text{rank}}_{i}} \end{array}$$ (16)
  where rank_*i*_ is the rank position of the first relevant document for query *i*. Queries for which no relevant document is retrieved within the evaluation cutoff contribute a reciprocal rank of 0 to the sum.
- **R-Precision**: Precision computed at a cutoff equal to the number of relevant documents *R* for each query described in Equation 17 as:
  $$\begin{array}{l} \text{R-Precision}=\frac{\left|\left\{\text{relevant docs}\right\}\cap \left\{\text{top-}R\text{retrieved}\right\}\right|}{R} \end{array}$$ (17)
- **NDCG@5**: Normalized Discounted Cumulative Gain evaluated at rank 5, used to evaluate the fine-grained ranking quality of retrieved text passages by penalizing relevant items that appear lower in the sequence. Equations 18 and 19 describes how NDCG@5 is computed. For a given query, the Discounted Cumulative Gain (DCG@5) is computed as:
  $$\begin{array}{l} \text{DCG}@5=\overset{5}{\underset{j=1}{\sum }}\frac{2^{{\text{rel}}_{j}}-1}{\log_{2}\left(j+1\right)} \end{array}$$ (18)
  where rel_*j*_∈{0, 1} is a binary relevance indicator denoting whether the text chunk at rank *j* matches a gold-standard reference passage. The metric is normalized by the Ideal DCG (IDCG@5), which represents the highest achievable DCG score generated by a perfect sorting of the candidate pool. The global metric is reported as the macro-averaged mean across the complete query set *Q*:
  $$\begin{array}{l} \text{NDCG}@5=\frac{1}{\left|Q\right|}\overset{\left|Q\right|}{\underset{i=1}{\sum }}\frac{{\text{DCG}}_{i}@5}{{\text{IDCG}}_{i}@5} \end{array}$$ (19)
- **Domain F1**: Measures the categorical alignment of medical specialties between the retrieved papers and the gold-standard reference set, assessing the retriever's contextual awareness of the query's broad clinical field and serving as a proxy metric for tracking *topic drift*. The domain labels are derived explicitly from the source journal classifications indexed directly within the PubMed/National Library of Medicine (NLM) directory, mapping directly to the distinct clinical categories illustrated in our domain distribution analysis (see Figure 2). To ensure reproducibility, the metric is evaluated as a macro-averaged query-level *F*_1_-score. For each query *i*∈*Q*, let $D_{i}^{*}$ denote the set of unique medical domains associated with the gold-standard documents, and let ${\hat{D}}_{i}$ denote the set of unique medical domains extracted from the top-*k* retrieved documents. Equation 20 describes the process of computing micro-level domain F1 and Equation 21 the global domain F-1. The query-level domain precision ($P_{i}^{\text{dom}}$), recall ($R_{i}^{\text{dom}}$), and *F*_1_-score ($F1_{i}^{\text{dom}}$) are defined as:
  $$\begin{array}{lll} P_{i}^{\text{dom}} & = & \frac{\left|{\hat{D}}_{i}\cap D_{i}^{*}\right|}{\left|{\hat{D}}_{i}\right|},\;R_{i}^{\text{dom}}=\frac{\left|{\hat{D}}_{i}\cap D_{i}^{*}\right|}{\left|D_{i}^{*}\right|},\\ F1_{i}^{\text{dom}} & = & \{\begin{array}{cc} \frac{2\cdot P_{i}^{\text{dom}}\cdot R_{i}^{\text{dom}}}{P_{i}^{\text{dom}}+R_{i}^{\text{dom}}} & \text{if}P_{i}^{\text{dom}}+R_{i}^{\text{dom}}>0\\ 0 & \text{otherwise} \end{array} \end{array}$$ (20)
  The global Domain *F*_1_ score reported in our evaluation tables is the macro-averaged mean computed across the entire query set:
  $$\begin{array}{l} \text{Domain F1}=\frac{1}{\left|Q\right|}\overset{\left|Q\right|}{\underset{i=1}{\sum }}F1_{i}^{\text{dom}} \end{array}$$ (21)
- **Figure metrics**: To evaluate the performance of the figure discovery pipeline, Figure Precision (*P*_fig_), Recall (*R*_fig_), and *F*_1_-score (*F*1_fig_) are reported as macro-averages computed across individual queries. For each figure-dependent query *i*∈*Q*_*F*_, metrics are calculated using the retrieved figure set ${\hat{F}}_{i}$ against the gold figure annotations $F_{i}^{*}$ Equations 22 and 23 describe this process:
  $$\begin{array}{lll} P_{i} & = & \frac{\left|{\hat{F}}_{i}\cap F_{i}^{*}\right|}{\left|{\hat{F}}_{i}\right|},\;R_{i}=\frac{\left|{\hat{F}}_{i}\cap F_{i}^{*}\right|}{\left|F_{i}^{*}\right|},\\ F1_{i} & = & \{\begin{array}{cc} \frac{2\cdot P_{i}\cdot R_{i}}{P_{i}+R_{i}} & \text{if}P_{i}+R_{i}>0\\ 0 & \text{otherwise} \end{array} \end{array}$$ (22)
  The global metrics reported in the evaluation tables are the macro-averaged means across the entire figure-dependent query set:
  $$\begin{array}{lll} P_{\text{fig}} & = & \frac{1}{\left|Q_{F}\right|}\overset{\left|Q_{F}\right|}{\underset{i=1}{\sum }}P_{i},\;R_{\text{fig}}=\frac{1}{\left|Q_{F}\right|}\overset{\left|Q_{F}\right|}{\underset{i=1}{\sum }}R_{i},\\ F1_{\text{fig}} & = & \frac{1}{\left|Q_{F}\right|}\overset{\left|Q_{F}\right|}{\underset{i=1}{\sum }}F1_{i} \end{array}$$ (23)
  **Figure hit rate** measures the proportion of figure-dependent queries for which at least one retrieved figure belongs to the gold annotation set, providing a query-level binary assessment of figure retrieval coverage Equation 24 describes this process as:
  $$\begin{array}{l} {\text{HitRate}}_{\text{fig}}=\frac{1}{\left|Q_{F}\right|}\overset{\left|Q_{F}\right|}{\underset{i=1}{\sum }}1\left[|{\hat{F}}_{i}\cap F_{i}^{*}|\geq 1\right] \end{array}$$ (24)
  where *Q*_*F*_ denotes the set of figure-dependent queries (|*Q*_*F*_| = 84) and **1**[·] is the indicator function.

##### 3.2.7.3 Evaluation design rationale

The evaluation framework adopted in this study is designed to assess a fundamentally different objective from the official BioASQ-13b task B competition. The competition evaluates document retrieval performance across all question types using Mean Average Precision (MAP) over PubMed article IDs and snippet-level F1 over extracted passage spans (Nentidis et al., 2026). Our work, by contrast, operates exclusively on summary-type questions and evaluates an end-to-end retrieval-generation pipeline over PMC full-text chunks—evaluation units that are structurally incompatible with competition-style MAP and snippet F1. BioASQ-13b is therefore employed in this study as a source of expert-annotated gold evidence and a rigorous question set, not as a competition benchmark. The retrieval metrics adopted in this study; P@5, R@10, MRR, and R-Precision, and our own domain-based metrics, are consistent with RAG-oriented retrieval evaluation literature (Gao et al., 2024), where ranked candidate quality and coverage directly determine generation input quality, and where exhaustive recall measured by MAP is neither applicable nor informative for a pipeline that selects a fixed context window for generation.

For generation quality, the official BioASQ-13b evaluation incorporates expert human assessment for linguistic and semantic coherence. Conducting human evaluation at the scale of our 650-question generation benchmark and 290-question retrieval evaluation set is operationally infeasible for a study of this scope—a constraint consistently acknowledged in contemporary large-scale biomedical NLP benchmarking (Liu et al., 2025). We therefore employ a multi-metric evaluation framework spanning lexical overlap (ROUGE-1, ROUGE-L, BLEU), structural similarity (GLEU), semantic alignment (METEOR, BERTScore F1), and domain-aware sentence-level similarity (SBERT cosine similarity via S-PubMedBERT-MS-MARCO). This combination ensures that evaluation captures both surface-level lexical precision and deep semantic fidelity, providing a robust and reproducible proxy for expert judgment that is consistent with established practice in large-scale biomedical summarization evaluation (Liu et al., 2025). The use of domain-adapted embeddings in both BERTScore and SBERT cosine similarity further ensures that biomedical terminology and clinical synonymy are handled appropriately, addressing a known limitation of general-purpose semantic metrics in the biomedical domain.

## 4 Results

In this section, we present the performance of the FFHRAG system across two experiments. We evaluate the system in two distinct phases to isolate the generator component from the retriever. We first identify the optimal Large Language Model (LLM) generator for biomedical summarization, before evaluating the retriever component end-to-end. The experiments were performed using Kaggle's Nvidia Tesla P100 GPU in a Kaggle Jupyter Notebook environment. It has a CPU RAM memory of 29 GB, 4 vCPU cores, a GPU memory of 16GB, 3,584 Cuda Cores, and a Memory Bandwidth of 732 GB/s.

### 4.1 Phase-1: Generator evaluation

The objective of Phase 1 was to establish a high-performance baseline for the generative stage. We selected a diverse cohort of six state-of-the-art large language models varying in parameter scale, context window capacity, and deployment infrastructure. The evaluation suite includes two high-performance API driven models: Meta AI's Groq Llama 3.1 8B Instruct, and Google's Gemini Flash 2.5. To complement these hosted service models, four open-weight models were evaluated using local infrastructure deployed in native FP16 precision using the vLLM inference engine. These include HuggingFace's Zephyr 7-B Beta, Mistral AI's Mistral 7-B instruct, Qwen's 2.5 7-B Instruct, and Microsoft's Phi-3 Mini. Models were assessed using a combination of lexical overlap, structural, and semantic metrics to ensure the generated answers remained both factually accurate and linguistically cohesive. The temperature and top_p were kept as 0.7 and 0.9 respectively for all models used in this experiment. The complete details of the LLM models used, including Parameter size, Context Window, Quantization, is described in Table 3.

**Table 3:** Architectural specifications and serving configurations of evaluated baseline generators (Phase-1).

Manuscript label	Exact API/repository identifier	Developer	Parameters	Context window	Quantization	Serving infrastructure
Groq Llama	meta-llama/Llama-3.1-8B-Instruct	Meta AI	8.03B	128,000 tokens	Mixed INT8/FP16	Groq Cloud API (LPU Array)
Gemini Flash 2.5	google/gemini-2.5-flash	Google DeepMind	Proprietary (MoE)	1,000,000 tokens	Undisclosed	Google AI Studio API (TPU Pods)
Zephyr	HuggingFaceH4/zephyr-7b-beta	Hugging Face	7.24B	4,000 tokens	Native FP16	Kaggle Notebook Environment
Mistral	mistralai/Mistral-7B-Instruct-v0.3	Mistral AI	7.24B	32,000 tokens	Native FP16	Kaggle Notebook Environment
Qwen	Qwen/Qwen2.5-7B-Instruct	Alibaba Cloud	7.61B	128,000 tokens	Native FP16	Kaggle Notebook Environment
Phi-Mini 3	microsoft/Phi-3-mini-128k-instruct	Microsoft	3.82B	128,000 tokens	Native FP16	Kaggle Notebook Environment

Table 4 clearly indicates that **Groq Llama** was the superior model among the others. It scored maximum on all the dimensions tested against it. This was reflected through the highest ROUGE-L score of 0.2459, METEOR score of 0.3022, and SBERT similarity of 0.8070. Such scores mean that the Groq Llama has the ability to sustain the semantic thread of the query in the complex clinical evidence extracted from various documents. High BERT Precision score of 0.8967 and Recall score of 0.8857 clearly reveal that Groq Llama not only gives coherent and comprehensive summaries but are also factually correct. **Gemini Flash 2.5** followed in second place as a strong performer, demonstrating proper structural coherence with a **ROUGE-L of 0.2331**, and a **Bert-Score F1 of 0.8610**, indicating that its coherence is comparable to Llama. However, Gemini's results across other metrics were consistently 2%–3% lower than Llama, making Llama as the undoubted winner, and as the ideal model choice for the FFHRAG pipeline.

**Table 4:** Phase 1 Generator performance benchmarking (650 questions).

Model	ROUGE-1	ROUGE-L	BERT P	BERT R	BERT F1	SBERT	BLEU	GLEU	METEOR
**Groq Llama**	**0.3859**	**0.2459**	**0.8967**	**0.8857**	**0.8911**	**0.8070**	**0.3051**	**0.2071**	**0.3022**
Gemini Flash 2.5	0.3577	0.2331	0.8600	0.8623	0.8610	0.7760	0.2739	0.1894	0.2848
Zephyr	0.3530	0.2448	0.8446	0.8443	0.8445	0.7718	0.2713	0.1803	0.2633
Mistral	0.3453	0.2205	0.8459	0.8522	0.8490	0.7402	0.2710	0.1781	0.2695
Qwen	0.3075	0.2116	0.8535	0.8589	0.8560	0.7391	0.2397	0.1599	0.2380
Phi-Mini 3	0.2272	0.1674	0.8300	0.8407	0.8352	0.6019	0.1902	0.1102	0.1720

The bolded text indicates the best performing generator, i.e. Groq Llama.

The other models showed a clear performance stratification, mainly due to model size and architecture. **Zephyr** and **Mistral** were both good performers in their intermediate range. Zephyr showed better structural similarity with a **ROUGE-L** score of 0.2448, which is actually higher than Gemini's, but performed poorly on semantic coherence (SBERT: 0.7718). **Qwen** displayed decent performance concerning semantic similarity (BERT F1: 0.8560), but fell short on lexical metrics (ROUGE-1: 0.3075), suggesting the model paraphrased abstract concepts without understanding the content retrieved with respect to the query. The most noticeable drop off in performance was demonstrated by **Phi-Mini 3**, achieving the lowest scores on GLEU (0.1102), METEOR (0.1720), and SBERT similarity (0.6019). This indicates that smaller models might lack the necessary parametric capacity to absorb the evidence provided and generate coherent summaries, especially in a technically dense domain such as biomedicine. These findings suggest that for a complex task like biomedical query summarization, a high-capacity model like Llama is required to effectively absorb the scattered evidence and generate a coherent summary from it.

### 4.2 Phase-2: Retrieval evaluation

We evaluate the impact of each component of the retriever by performing a rigorous ablation study of the FFHRAG. We start from the vanilla RAG and move onto the Figure-First Retriever, assessing the performance improvement made by the addition of each individual component. Each variant is executed using three distinct random seeds—42, 43, and 44, to ensure statistical reliability and guard against initialization bias. By evaluating the pipeline across multiple seeds, we mitigate the impact of stochastic variations inherent in dense vector indexing, embedding alignments, and generative sampling. This multi-seed validation framework ensures that the empirical performance gains observed throughout the ablation stages are robust, stable, and completely reproducible, rather than artifacts of a single favorable initialization state. Table 5 reflects the detailed performance of the ablation-study, and Table 6 reflects the performance of the variants in our own domain-based metrics. It is to be noted that while the set of top-20 chunks are completely used for the retrieval evaluation metrics, the number of chunks passed as input to the generator varies per variant, and is discussed in Table 1.

**Table 5:** Ablation study results across retrieval and generation metrics (290 questions).

Variant	P@5	R@10	MRR	R-Prec	NDCG@5	ROUGE-1	ROUGE-L	Bert-Score	SBERT
Vanilla	0.2993 ± 0.0000	0.6933 ± 0.0000	0.7543 ± 0.0000	0.5903 ± 0.0000	0.7480 ± 0.0000	0.3824 ± 0.0020	0.2451 ± 0.0028	0.8183 ± 0.0011	0.9721 ± 0.0003
BM25 Hybrid	0.3138 ± 0.0060	0.7322 ± 0.0154	0.7618 ± 0.0156	0.5959 ± 0.0166	0.7852 ± 0.0192	0.3849 ± 0.0046	0.2562 ± 0.0032	0.8257 ± 0.0161	0.9726 ± 0.0015
CE Rerank	0.3611 ± 0.0004	0.7991 ± 0.0000	0.8331 ± 0.0018	0.6916 ± 0.0010	0.8914 ± 0.0008	0.4068 ± 0.0037	0.2623 ± 0.0018	0.8326 ± 0.0006	0.9748 ± 0.0002
Hierarchical	0.3607 ± 0.0000	0.7991 ± 0.0000	0.8310 ± 0.0000	0.6905 ± 0.0000	0.8908 ± 0.0000	0.3481 ± 0.0055	0.2205 ± 0.0019	0.8182 ± 0.0009	0.9676 ± 0.0002
FigureBoost	0.3615 ± 0.0004	0.7991 ± 0.0000	0.8358 ± 0.0018	0.6950 ± 0.0010	0.8921 ± 0.0008	0.4070 ± 0.0029	0.2618 ± 0.0003	0.8324 ± 0.0005	0.9748 ± 0.0004
**FFHRAG**	**0.3616** ± 0.0008	**0.7991** ± 0.0000	**0.8358** ± 0.0018	**0.6951** ± 0.0010	**0.8926** ± 0.0008	**0.4137** ± 0.0036	**0.2678** ± 0.0016	**0.8326** ± 0.0003	**0.9748** ± 0.0002

The bolded value here indicates the performance of the complete FFHRAG architecture.

**Table 6:** Domain metrics.

Variant	Domain-Prec	Domain-Recall	Domain-F1
Vanilla	0.4864 ± 0.0000	0.8600 ± 0.0000	0.5679 ± 0.0000
BM25 Hybrid	0.4834 ± 0.0000	0.8745 ± 0.0000	0.5688 ± 0.0008
CE Rerank	0.6060 ± 0.0003	0.8440 ± 0.0000	0.6559 ± 0.0003
Hierarchical	0.6051 ± 0.0010	0.8440 ± 0.0000	0.6554 ± 0.0003
FigureBoost	0.6074 ± 0.0010	0.8440 ± 0.0000	0.6573 ± 0.0007
FFHRAG	0.6074 ± 0.0000	0.8440 ± 0.0000	0.6573 ± 0.0007

#### 4.2.1 Vanilla retriever

The Vanilla RAG serves as the foundational baseline for the FFHRAG pipeline, implementing a standard single-stage dense retrieval approach. In this configuration, the query gets encoded into a 768-dimension vector using the *S-PubMedBERT-MS-MARCO* bi-encoder. The system effectively performs a cosine similarity search across the entire corpus of 1,101,618 chunks present in the FAISS index, due to the embeddings being L2-normalized. The retriever extracts a candidate pool of the top 60 chunks, which are then de-duplicated and ordered based on their similarity scores. From this refined pool, the top-10 chunks are selected as input for the generator.

The quantitative results underscore the performance boundaries of relying exclusively on global semantic bi-encoding within a highly dense biomedical corpus. Because vector search is entirely deterministic, the retrieval metrics exhibit zero variance across the three experimental seeds, yielding a **P@5** of 0.2993, a **R@10** of 0.6933, and a **MRR** of 0.7543. While the retriever also shows reasonable contextual positioning with a **Domain F1** score of 0.5679, it's fine grained chunk prioritization remains low. This ranking challenge directly impacts downstream generation. The generator achieves a **ROUGE-1** of 0.3824 ± 0.0028, and a **BERT-Score** of 0.8183. A **SBERT Cosine** score of 0.9721 ± 0.0003 indicates that the summaries constructed are semantically fluent, however, its specific evidential accuracy is capped by the lack of fine-grained ranking precision, establishing a clear empirical baseline that subsequent hybrid and cross-modal variants are designed to improve upon.

#### 4.2.2 BM-25 Hybrid

The BM25 Hybrid variant marks our first major architectural shift by introducing lexical signal integration. The vanilla baseline relies purely on dense semantic embeddings, failing on retrieving the exact technical keywords and chunks required for biomedical precision. To address this limitation, the BM25 Retriever implements a parallel sparse search using a BM25s index. At query time, the system retrieves 100 candidates from both the FAISS dense index, and the BM25s sparse index. These two separate candidate lists are then unified via **Reciprocal Rank Fusion**, which assigns a combined score to each chunk based on its rank in both the dense and sparse results as described in Equation 25:

$$\begin{array}{l} s_{\text{RRF}}\left(d\right)=\underset{r\in R}{\sum }\frac{1}{k+r\left(d\right)} \end{array}$$ (25)

where *k* = 60 is a standard constant and r(d) is the rank of document d.

The impact of this hybrid retrieval approach is clearly visible. By anchoring retrieval in lexical evidence, the **Precision@5** improves from the Vanilla baseline of 0.2993 to 0.3138 ± 0.0060, while the **MRR** increases from 0.7543 to 0.7618 ± 0.0156. This improvement confirms that exact terminology is a critical discovery signal in the biomedical domain. This superior retrieval quality has a direct impact in the generation, with **ROUGE-L** rising to 0.2562 ± 0.0032 and **Bert-Score** improving to 0.8257 ± 0.0161. This hybrid variant confirms that exact lexical grounding of queries is a key requirement for biomedical generation performance.

#### 4.2.3 Cross-encoder reranking

The Cross-Encoder Reranking variant builds directly upon the BM25 Hybrid pipeline by introducing a deep semantic re-scoring and context diversification layer. While the hybrid fusion of dense and sparse signals significantly improved retrieval precision, the RRF-fused candidate pool still relies on bi-encoder and BM25s scores, both of which evaluate query and chunk representation independently. To bridge this gap, this variant introduces a specialized transformer-based cross-encoder, ncbi-MedCPT-Cross-Encoder, which performs joint token-level modeling. By concatenating the query and a candidate text chunk into a single input sequence, the cross-encoder's deep full-attention layers capture explicit token-to-token dependencies and fine-grained contextual relationships that independent vector projections inherently miss. The pipeline feeds the top-100 candidates surfaced by the hybrid RRF sequence directly into the cross-encoder to compute a precise logit relevance score for each pair. Any candidate chunk returning a logit score ≤ 0 is discarded as irrelevant. The surviving candidates are then passed through **Maximal Marginal Relevance** (MMR) with λ = 0.6, which iteratively selects the top-20 chunks that balance retrieval relevance against inter-chunk semantic redundancy, from the CE-filtered candidate pool, preventing passages from the same document from dominating the final context window.

The addition of cross-encoder and MMR provides measurable gains over the BM25 hybrid across both retrieval and generation dimensions. **Precision@5** improves to 0.3611 ± 0.0004 while **MRR** improves to 0.8331 ± 0.0018, showcasing that cross-encoder preserves the strong lexical precision introduced by the hybrid stage. The quality of generation metrics improve as well, with **ROUGE-L** going from 0.2562 to 0.2623 ± 0.0018, **ROUGE-1** going from 0.3849 to 0.4068 ± 0.0037, and Bert-Score going from 0.8257 to 0.8326 ± 0.0006. This shows that CE-reranked, MMR-diversified context gives the generator a richer and less redundant set of evidence. These results establish the CE Reranking variant as a strong text-only baseline, demonstrating that Cross Encoder based reranking and scoring is a necessary precursor for Retrieval in Biomedical domains.

#### 4.2.4 Hierarchical retriever

The Hierarchical variant builds on top of the Re-rank approach and adds a **section expansion** module following the MMR selection process. Instead of sending the selected chunks straight to the generator, the model identifies the parent section corresponding to each chunk and replaces it with the full content of the section trimmed down to 2,500 characters and anchored on the most important chunk. The intention behind this design choice is to provide better contextual context by including the discussion and evidence related to the chunk in question.

However, the empirical results reveal a consistent performance degradation across all generation metrics, compared to the Re-rank baseline. **ROUGE-L** drops from 0.2623 to 0.2205 and **Bert-Score** decreases from 0.8326 to 0.8182, while retrieval metrics remain largely unchanged. This decline is attributable to a structural asymmetry between section expansion and chunk-level retrieval. In the Hierarchical variant, each of the four context slots is occupied by a full section passage of up to 2,500 characters, meaning the generator receives fewer but larger evidence units—the surrounding section text, while contextually adjacent, is not guaranteed to be query-relevant and introduces noise that dilutes the high-precision signal produced by the cross-encoder. This suggests that for biomedical summarization at BioASQ ideal-answer length, the density of query-relevant signal per token matters more than contextual continuity, and that section-level expansion trades precision for coverage in a way that is net negative for generation quality. The Hierarchical variant is therefore excluded from the primary comparison in favor of the chunk-level pipeline, which is carried forward in subsequent variants. The character limit has also been reduced to 1,500, which was used by the rerank variant for the upcoming variants, balancing sufficient context for abstractive generation with retrieval precision.

#### 4.2.5 Figure boost

The Figureboost variant introduces the core figure-first signal principle of the FFHRAG pipeline, as described in Section 3.1.2. Due to the performance dip observed in the Hierarchical variant, the FigureBoost and the subsequent FFHRAG replicates the Re-Rank architecture, with the sole addition of the figure-first retrieval. Critically, while the figure-captions are retrieved and evaluated, they are **not** injected into the generator prompt in this variant, isolating the retrieval impact of the figure signal from any generative benefit.

The introduction of the figure-first signal produces measurable improvements over the Re-rank baseline across the retrieval metrics. **R-Precision** goes up from 0.6916 to 0.6950 ± 0.0010 and **Precision@5** goes up from 0.3611 to 0.3615 ± 0.0004 This shows that the figure boost does bring up more relevant documents in the candidate pool. The quality of the generation remains almost the same however. This proves that while the retrieval process is enhanced by the figure-guided architecture, the generator still lacks the information and the context which the injection of the figure caption brings, which can be observed in the upcoming variant.

#### 4.2.6 Figure-First Hybrid RAG

The full FFHRAG variant is architecturally similar to the Figureboost variant in its retrieval pipeline. However, the retrieved figure-captions are injected into the generator as well, along with the text chunks. This creates a clean controlled comparison between the two variants: any difference in generation metrics is attributable exclusively to the figure-captions injected, while the retrieval metrics are expected to remain similar, or even identical. The marginal differences observed in the retrieval metrics are attributable to non-deterministic floating-point ordering in the IVFFlat FAISS index across evaluation runs, rather than any architectural divergence.

The results confirm this separation. Retrieval metrics remain near identical, between the two variants. However, there is a clear improvement from the caption injection: **ROUGE-1** improves from 0.4070 to 0.4137 ± 0.0036, **ROUGE-L** from 0.2618 to 0.2678 ± 0.0016, and **BERTScore** from 0.8324 to 0.8326 ± 0.0003. These gains demonstrate that figure-captions carry complementary information, beyond what the retrieved text chunks provide. The figure-captions introduce domain-specific mechanistic detail that directly improves the factual density of retrieved evidence, resulting in improved generation quality.

Notably, it should be pointed out that although the absolute value of Precision@5 (0.3616) of FFHRAG is seemingly less than the value of Recall@10 (0.7991), this difference is a result of the particular structure of the system oriented toward high recall. Precision@5 considers the relevance density in the top-5 retrieved fragments and is usually informative for systems returning results with very high precision. In FFHRAG, on the contrary, recall orientation is implemented to provide the generator with sufficient amount of evidence. In biomedical query summarization, recovering necessary evidence across multiple documents and synthesizing it into a cohesive paragraph-length answer is the priority. This effectiveness is confirmed by the significantly higher R-Precision of 0.6951, which demonstrates that the system successfully recovers and ranks relevant evidence when the evaluation cutoff is matched to the actual number of gold documents available for each query. Consequently, R-Precision provides a more representative measure of retrieval quality for this architecture, while Precision@5 is reported primarily for completeness and standard cross-system comparability.

### 4.3 Minimal character prompt budget performance analysis

To test the performance of our architecture under extreme contextual constraints, we evaluated the core pipeline variants with a prompt budget of 900 characters, as compared to the regular 1,500 character used for the core variants. We decided that a budget of 900 characters would be required at worst in order to absorb the caption injection on top of the context provided by the text chunks as well. The full results of the prompt budgeted run is presented in Table 7. Across the three experimental seeds, the **CE Rerank** variant produced a competitive initial baseline, producing a ROUGE-1 score of 0.4039 ± 0.0016, a ROUGE-L score of 0.2599 ± 0.0025, and a BertScore of 0.8324 ± 0.0003. When introducing our fine-grained visual retrieval guidance layer, the standalone FigureBoost variant yields an incremental performance lift, advancing ROUGE-1 to 0.4041 ± 0.0006 and ROUGE-L to 0.2603 ± 0.0006. Crucially, FigureBoost demonstrates a noticeable reduction in variance across the iterations, suggesting that cross-modal caption-to-chunk matching provides a highly stable anchoring mechanism when the available textual token budget is limited. The fully unified framework, FFHRAG, achieves the best performance under these conditions, improving the lexical overlap metrics to a ROUGE-1 score of 0.4074 ± 0.0005 and a ROUGE-L score of 0.2618 ± 0.0004, alongside an optimal BertScore of 0.8330 ± 0.0001. Across all three configurations, the SBERT Cosine similarity remains remarkably tightly bound within a narrow margin from 0.9747 to 0.9750, indicating that generic semantic fluency remains uniform across the configurations while specific factual coverage and precise wording are optimized by the integrated cross-modal pipeline. This prompt-budgeted analysis proves that the performance of our metrics remains competitive, even under strict context boundaries. The structural interplay of domain-calibrated re-ranking and continuous visual grounding allows the model to extract and synthesize highly concentrated biomedical evidence effectively.

**Table 7:** Generation metrics for 900 char prompt budget.

Variant	ROUGE-1	ROUGE-L	BertScore	SBERT Cosine
CE Rerank	0.4039 ± 0.0016	0.2599 ± 0.0025	0.8324 ± 0.0003	0.9750 ± 0.0001
FigureBoost	0.4041 ± 0.0006	0.2603 ± 0.0006	0.8320 ± 0.0001	0.9747 ± 0.0001
FFHRAG	0.4074 ± 0.0005	0.2618 ± 0.0004	0.8330 ± 0.0001	0.9748 ± 0.0002

### 4.4 Latency and efficacy analysis

In this section, we analyze the operational viability and infrastructure overhead of the FFHRAG framework, and its subsequent variants. The system execution parameters are decoupled into retrieval-phase latency, end-to-end total latency, and peak GPU VRAM consumption. The complete performance metrics for the variants for all seeds is provided in Table 8.

**Table 8:** Computational telemetry and latency metrics.

Variant	Latency Retrieval median	Latency Retrieval p95	Latency Total Median	Latency Total P95	Peak GPU VRAM MB
Vanilla	36.4015 ± 1.2367	43.1347 ± 2.1389	15,379.8745 ± 26.9238	18,560.1438 ± 21.4234	2,680.4736 ± 0.0000
BM25 Hybrid	354.4931 ± 17.8973	391.3844 ± 19.3363	14,870.4858 ± 91.2620	23,225.8197 ± 74.7987	2,680.4736 ± 0.0000
CE Rerank	910.5092 ± 26.7692	1,130.5452 ± 35.1906	22,181.6554 ± 653.5080	28,930.5468 ± 327.6046	3,380.0288 ± 0.0000
FigureBoost	1,481.4739 ± 34.4471	2,146.7397 ± 21.1389	22,607.0017 ± 260.9913	29,638.8212 ± 241.6022	3,677.2729 ± 0.0000
FFHRAG	1,477.4739 ± 32.5432	2,089.7024 ± 49.9384	22,793.2092 ± 279.6315	29,844.5439 ± 31.5141	3,677.2729 ± 0.0000

The retrieval phase shows a predictable, step-by-step increment in computational latency as advanced re-ranking and multi-modal alignment layers are introduced into the pipeline. The **Vanilla** dense retrieval configuration operates at an ultra low latency baseline, only taking about 36.40 ms to retrieve information for a query. The addition of BM25 sparse indexing channel in the **BM25** variant shifts the median retrieval latency to 354.49 ms, nearly 10 times more than that of the vanilla variant. This represents the overhead of executing parallel asynchronous text lookups across distinct index formats and running the RRF algorithm to merge the top candidate arrays. The Peak GPU VRAM of both the Vanilla and the BM25 variant remains constant at 2.6 GB. This is because we load the dataset, which contains both dense and sparse embeddings together for each and every variant. The **Cross-Encoder Reranking** variant further increases the median latency to about 910.50 ms. This jump isolates the computational cost of executing the joint similarity measurement of the query and caption pairs by the cross-encoder over the top 100 RRF candidates. The GPU VRAM increases to 3.3 GB, which can be attributed to the addition of the cross-encoder. The inclusion of visual signals in the **FigureBoost** and **FFHRAG** variants defines the upper boundary of retrieval latency, centering at around 1,478 ms. The added 560 ms compared to the rerank variant represents the dual-encoder text-to-caption embedding generation and the subsequent localized similarity matrix computations required to calculate fine-grained visual boosts. The Peak GPU VRAM also increases from 3.3 GB to 3.6 GB, which represents the inclusion of the additional figure and figure caption embeddings to the dataset loaded in memory.

Regarding the Generation metrics, a critical trend emerges when evaluating total end-to-end execution times. Across all core configurations, total latency is fundamentally dominated by the auto-regressive generation phase of the downstream LLM, accounting for over 93% of the overall execution footprint. Total median execution time sits at around 15 s for the Vanilla and BM25 variants, and around 23 s for the advanced CE Rerank, FigureBoost, and FFHRAG variants. This latency shift is primarily driven by the quality and density of the retrieved context. While the baseline models often pass less aligned, sparser context sets, the advanced variants leverage cross-encoders and visual boost anchoring to pack the context window with highly granular, information-rich text segments. Processing this dense evidence rich chunks slightly lengthens the model's token attention and decoding cycles, resulting in the longer generation time in the advanced variants.

### 4.5 Figure-Caption contribution: subset analysis

In this section, we aim to answer the following question: “Does the retrieval and injection of figure-captions for questions which do not require figures have a negative impact?” In order to perform this analysis, we divide the subset of 290 questions used for the phase-2 evaluation into two categories: 84 questions where figures and captions may have a positive impact, and 206 questions which may not necessarily require figures. The former consists of in-depth queries such as “Explain the pathway of a gene RNA function,” where a visual diagram and its corresponding caption describing the structure and flow of a gene may provide key additional evidence to a generator in minimal token size, while the latter consists of fact-checking or short-answer type questions, which can be answered sufficiently with textual evidence alone. We perform our generation quality analysis for the Rerank, Figureboost and FFHRAG variants on both of these subsets. The metrics are available in Table 9.

**Table 9:** Generation metrics by figure dependency subset.

Variant	Subset	ROUGE-1	ROUGE-L	BERT-Score	SBERT Cosine	Caption Survival
CE Rerank (Baseline)	Fig-True (*n* = 84)	0.3893	0.2372	0.8316	0.9748	–
CE Rerank (Baseline)	Fig-False (*n* = 206)	0.4140	0.2726	0.8330	0.9748	–
FigureBoost (Δ)	Fig-True (*n* = 84)	−0.0002	+0.0008	−0.0010	−0.0002	20%
FigureBoost (Δ)	Fig-False (*n* = 206)	+0.0002	−0.0011	+0.0001	0.0000	12%
**FFHRAG** (**Δ**)	**Fig-True** (*n* = **84**)	**+0.0181**	**+0.0167**	−0.0002	−0.0002	**20%**
**FFHRAG** (**Δ**)	**Fig-False** (*n* = **206**)	**+0.0022**	**+0.0010**	0.0000	0.0000	**12%**

The bolded values here indicate the performances of the FFHRAG variant in the Fig-True and Fig-False subsets.

Table 9 reveals two important findings. First, caption injection in FFHRAG improves generation quality on the *Fig-True* subset across ROUGE-1 and ROUGE-L metrics, compared to the ReRank baseline, with **ROUGE-L** increasing by 0.0167 and **ROUGE-1** by 0.0181, and semantic alignment metrics (BERT-Score and SBERT) remain essentially stable (Δ ≤ 0.0002), confirming that figure-captions carry complementary evidence that enriches responses to visually grounded biomedical queries. These gains can solely be attributed to the injection of figure-captions, as the FigureBoost variant shows no major gains across these metrics in the Figure-True subset. Second, and more importantly, figure-first retrieval and caption-injection does not negatively impact generation performance on the *Fig-False* subset. FFHRAG outperforms the rerank variant marginally here, the metrics remain largely similar, meaning that even with caption injection, no irrelevant captions are fed to the generator which decreases the generation quality. The Caption Survival for Fig-False questions is 12%, meaning that 88% of captions considered as noise is filtered out successfully by the cross-encoder, before being sent to the generator. This indicates that the cross-encoder-filtered captions injected into the prompt are sufficiently relevant that they do not introduce noise even for questions that do not inherently require visual evidence, directly answering the motivating question of this section in the negative. The generation metrics being higher on both FFHRAG and the FigureBoost variant for the Figure-False subset is due to the fact that Figure-False subset contains simpler queries that require short answers, compared to the descriptive, longer answers required for the Figure-True questions.

Table 10 evaluates the quality of the four-channel figure retrieval stage against gold figure annotations on the *Fig-True* subset. Figure retrieval metrics are reported exclusively for FFHRAG, as FigureBoost utilizes the same figure retrieval mechanism as the FFHRAG, and also uses figures solely as an internal boosting signal without exposing them as retrievable outputs—rendering precision and recall against gold annotations a category error for that variant. FFHRAG achieves a **Figure Recall** of 0.7599 and a **Figure HitRate** of 0.9286, indicating that the system successfully surfaces at least one gold-annotated figure for nearly 93% of figure-dependent queries. The lower Figure Precision (0.6210) compared to Recall (0.7599) is expected given the retrieval design; the four-channel pipeline is intentionally recall-oriented, prioritizing coverage of relevant figures over strict precision, with the cross-encoder performing final selection for prompt injection. While reported for completeness, we intentionally temper the strength of our figure-specific claims, acknowledging that the absence of independent inter-annotator agreement or parallel specialist verification makes absolute metric validation difficult to fully guarantee.

**Table 10:** Figure retrieval metrics on Fig-True subset (*n*=84).

Variant	Fig-P	Fig-R	Fig-F1	Fig-HitRate
FFHRAG	0.6210	0.7599	0.6573	0.9286

### 4.6 Hyperparameter sensitivity and stability analysis

To validate the robustness of the chosen default hyperparameter configuration, we perform a sensitivity sweep over four key parameters: MMR diversity weight λ, FigureBoost score amplification factor β, FigureBoost threshold τ, and RRF offset constant *k*, evaluating each against P@5, R@10, MRR, and R-Precision on the full 290-question Phase-2 evaluation set. The results are visualized in Figure 5.

**Figure 5:**
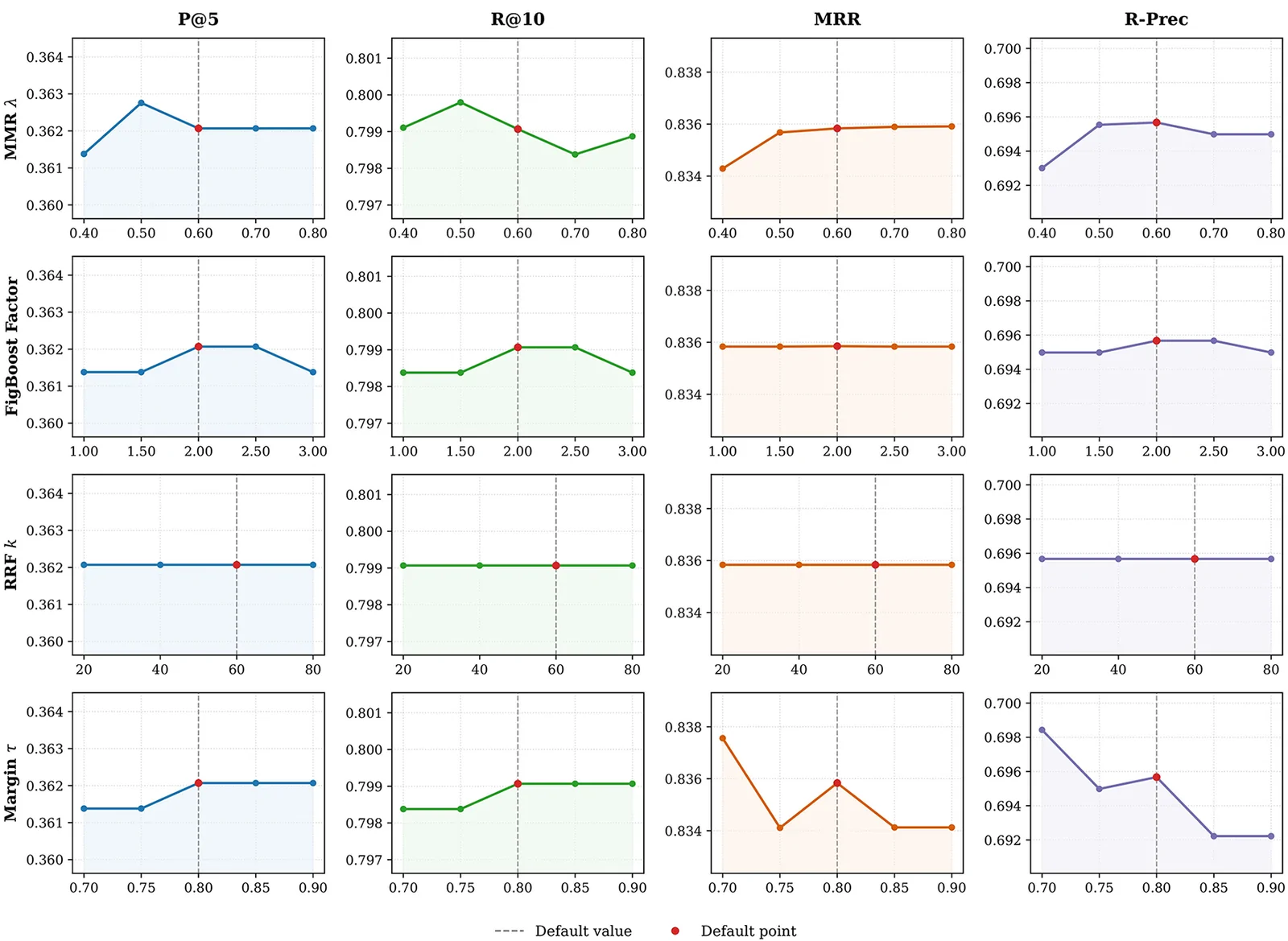
Hyperparameter sensitivity analysis for FFHRAG across four parameters (MMR λ, FigureBoost Factor, RRF *k*, and Threshold Margin τ) and four retrieval metrics. Dashed vertical lines indicate the chosen default value; red points mark the corresponding performance at the default.

The sweep reveals that FFHRAG is largely insensitive to changes in all four parameters. For MMR λ, varying the diversity-relevance trade-off from 0.4 to 0.8 produces a P@5 range of only 0.3613 to 0.3627, with MRR remaining virtually unchanged at 0.8356 to 0.8358 across the entire range. The default value of λ = 0.6 sits at the performance plateau, confirming that modest diversity injection does not sacrifice relevance. For the FigureBoost factor, values below 2.0 yield marginal reductions in both P@5 and R@10, suggesting that a boosting factor below 2.0 provides insufficient signal to meaningfully re-rank figure-bearing passages within the candidate pool, while values above 2.0 begin to over-promote figures at the expense of purely textual candidates, causing a gradual decline in P@5 from 0.3620 to 0.3613 and R@10 from 0.7991 to 0.7983. The default of 2.0 thus represents the optimal amplification point. For the RRF offset *k*, performance is stable across *k*∈{20, 40, 60, 80}, with P@5 and MRR remaining effectively flat; *k* = 60 is chosen as the default for consistency with established practice. The threshold margin of τ_margin_ = 0.8 represents the optimal value for calculating figure caption relevance, metrics for values above 0.8 remain the same, but for values below the threshold, the results marginally drop across all metrics, suggesting that a lower threshold score boosts noisy chunks which degrade retrieval performance. Taken together, these results confirm that the reported Phase-2 performance reflects a genuinely robust configuration rather than one tuned to a narrow optimum.

### 4.7 Statistical implications

We assessed the statistical significance of the differences in generation quality between the CE Reranking, FigureBoost, and FFHRAG variants using the non-parametric, one-sided Wilcoxon signed-rank test (Woolson, 2008) on per-question scores across all 290 evaluation questions. To counteract Type I error inflation over highly correlated text-matching dimensions (ROUGE-1, ROUGE-L, BERTScore, and SBERT Cosine), we implement a global multiplicity adjustment strategy using the Holm procedure. We provide a singular Holm-adjusted *p*-value (*p*_Holm_) applying to each specific comparison and note that an optimal value of *p*_Holm_ < 0.05 indicates statistically significant results. This whole technique adheres to Family-Wise Error Rate (FWER) of α = 0.05 throughout the pool of *m* = 12 concurrent tests related to 4 generation metrics in 3 pairwise variants. The results of non-parametric estimates of localized effect size are provided as $r=|Z|/\sqrt{N}$. Additionally, bootstrap confidence intervals via 10,000 empirical resamples were computed to precisely quantify the statistical distribution, magnitude, and precision of the observed cross-modal improvements. All reported performance differences represent median per-question paired differences evaluated across the query distributions from the Wilcoxon test, and not the differences of global means. On the full evaluation set, FFHRAG demonstrates statistically significant improvements over the cross-encoder filtering baseline across ROUGE-1 (Δ = +0.012, *p*_Holm_ = 0.045, *r* = 0.147), and ROUGE-L (Δ = +0.010, *p*_Holm_ = 0.004, *r* = 0.164). Bootstrap confidence intervals confirm that none of these gains cross zero: ROUGE-1 [+0.004, +0.020], and ROUGE-L [+0.003, +0.018]. Furthermore, a McNemar test on ROUGE-L win/tie/loss margins indicates FFHRAG consistently outperforms the baseline on a per-query basis (43.6% wins vs. 26.5% losses, *p* = 0.009). SBERT cosine similarity showed no significant variation across systems (all *p*>0.05), indicating that all variants produce comparably fluent biomedical text regardless of retrieval strategy.

Stratified analysis on the 84 figure-relevant questions reveals amplified and sharper performance improvements: ROUGE-L increases to Δ = +0.018 (*p*_Holm_ = 0.007, *r* = 0.27) and ROUGE-1 to Δ = +0.021 (*p*_Holm_ = 0.04, *r* = 0.227), with both successfully surviving Holm correction and displaying strictly positive bootstrap confidence intervals. The larger standardized effect sizes observed on this subset compared to the lower baseline effects (*r* = 0.147 and *r* = 0.164) captured on the full evaluation set are consistent with our core figure-first design hypothesis: prompt-level caption injection provides its greatest operational benefit precisely on queries where visual evidence is highly informative. Ablation analysis further isolates caption injection as the primary generative driver within the cross-modal framework. FigureBoost, which applies figure-guided chunk boosting within the retrieval phase without injecting raw text captions into the generator prompt, produces no significant generation improvement over the CE Reranking baseline on any metric. By contrast, FFHRAG, which layers caption injection directly on top of the FigureBoost retrieval architecture, yields consistent improvements across ROUGE-1 and ROUGE-L over FigureBoost on these figure-relevant questions, as shown in Table 9. These results support our initial hypothesis, that the downstream generative utility of the figure-first pipeline is fundamentally attributable to prompt-level contextual grounding rather than cross-modal retrieval re-weighting alone.

## 5 Discussion

The experimental results presented across both evaluation phases demonstrate that augmenting a RAG pipeline with structured figure-caption retrieval yields consistent and measurable improvements in both retrieval and generation quality. In this section, we will discuss the broader implications of these findings for multimodal biomedical information retrieval, and reflect on the current limitations of the proposed system, and direction for future investigations.

### 5.1 Implications

The two phase evaluation approach of this work is purposeful. The first phase is a comprehensive analysis of generator selection, evaluating several different LLMs for determining which generates more effectively using retrieval context. By deciding on the generator in advance, it enables the second phase to isolate changes due to retrieval architecture as the reason for any difference in generation performance, since generator selection will remain constant. This approach is not only replicable but necessary methodologically; an inadequate generator might conceal gains in retrieval performance when considering end-to-end metrics.

A more practically significant discovery is the subset analysis in Table 9. Figure-based retrieval of text chunks and injection of figure-captions do not adversely affect generation quality even for questions that do not inherently require visual evidence. This implies that a classification gate, a component that would predict whether a query needs visual evidence or not is unnecessary in the current design. The filtering performed by the cross-encoder itself is sufficient for fact-based or short-answer queries; low-scoring captions are naturally deprioritized during prompt construction, while for pathway or mechanism-based queries, high-scoring captions surface and contribute meaningful structural evidence. This emergent gating behavior simplifies deployment considerably. This can be observed by the caption survival rate for Figure-False questions being lower than the Figure-True questions.

On a wider note, these findings highlight that there is a systematic gap in existing biomedical RAG systems. Biomedical literature is figure-dense by nature and pathway diagrams, structure illustrations, and experimental result plots carry information that is either absent, or only partially conveyed through text. Systems that operate exclusively on text discard this evidence. FFHRAG demonstrates that even without utilizing a multimodal generator component, and injecting only figure-captions into a text-based generator shows improvements, suggesting that caption-aware indexing has the potential to become a vital component for future biomedical retrieval systems.

### 5.2 Limitations and future work

Our study is constrained by three limitations. First, the evaluation is bounded by its dataset scope. All experiments are conducted on BioASQ-derived question sets, which predominantly reflect well-informed, research-oriented biomedical queries drawn from PubMed literature. How FFHRAG performs on noisier, less structured query types such as clinical decision support queries is an open question. Generalization to other biomedical document types, such as clinical trial reports, radiology notes, electronic health records, or non-English documents as evidence for retrieval remains unvalidated.

Secondly, a more fundamental limitation lies in the scope of the generator component. While the four-channel retrieval pipeline successfully surfaces both figure-captions as well as images, and uses them as an upstream signal to guide retrieval of textual evidence, the generator operates purely in text modality. This design choice was intentional as direct injection of raw figures into a generator introduces cross-modal alignment noise that could potentially affect generation performance for the worse. However, for a domain such as biomedicine, incorporation of figures directly into the generator is vital. Therefore, incorporating a multimodal generation component serves as a key direction for potential future work on this topic.

Third, while the manual annotation protocol adopted for the Phase-2 evaluation set represents the most defensible approach available in the absence of existing figure-level gold standards for BioASQ summary questions, the figure gold labels reflect the judgments of this study's authors rather than independent domain experts. Future work should engage specialist annotators to validate figure relevance judgments, particularly for highly specialized sub-fields such as genomics and oncology, which constitute the majority of the corpus, and to establish inter-annotator agreement scores as a formal, reliable measure for multimodal biomedical benchmarking.

## 6 Conclusion

Biomedical literature is inherently multimodal, yet existing RAG systems treat documents as purely textual, discarding the information encoded in figures, pathway diagrams, and experimental visualizations. This work presented Figure-First Hybrid RAG, an architectural paradigm that directly addresses this gap through figure-guided text retrieval. A four-channel retrieval pipeline selects the relevant figures first, and the text chunks which are related to the figures are given a boosting score, with a cross-encoder re-ranking selecting the most relevant text chunks and figure-captions for prompt injection. Through our two-phase evaluation, we were able to empirically demonstrate that figure based retrieval and injection of figure-captions improved both the generation and retrieval performance. These results establish figure-caption-aware retrieval as a practical, low-overhead augmentation to a biomedical RAG pipeline, one that improves generation quality without introducing cross-modality noise. We hope that this work encourages broader adoption of caption-aware indexing as a standard component in biomedical retrieval systems, and laying a foundation for future work integrating multimodal generation with structured figure evidence.

## Data Availability

The datasets presented in this study can be found in online repositories. The names of the repository/repositories and accession number(s) can be found below: https://github.com/rkv2005/Figure-Guided-Retrieval-for-Biomedical-Query-Summarization.
